# The fly route of extended-spectrum-β-lactamase-producing *Enterobacteriaceae* dissemination in a cattle farm: from the ecosystem to the molecular scale

**DOI:** 10.3389/frabi.2024.1367936

**Published:** 2024-04-10

**Authors:** Alann Caderhoussin, David Couvin, Gaëlle Gruel, Isaure Quétel, Matthieu Pot, Rémy Arquet, Alexis Dereeper, Jean-Christophe Bambou, Antoine Talarmin, Séverine Ferdinand

**Affiliations:** ^1^ Transmission, Reservoir and Diversity of Pathogens Unit, Institut Pasteur, Les Abymes, France; ^2^ Tropical Platform for Animal Experiments, Institut National de Recherche pour l'Agriculture, l'Alimentation et l'Environnement (INRAE), Le Moule, France; ^3^ Tropical Agroecology, Genetics and Livestock Systems Research Unit, Institut National de Recherche pour l'Agriculture, l'Alimentation et l'Environnement (INRAE), Petit-Bourg, France

**Keywords:** ESBL, *Enterobacteriaceae*, ST3268, oxytetracycline, selective pressure

## Abstract

**Introduction:**

This study aimed to understand the origin and to explain the maintenance of extended-spectrum β-lactamase (ESBL) *Enterobacteriaceae* isolated from food-producing animals in a third-generation cephalosporin (3GC)-free farm.

**Methods:**

Culture and molecular approaches were used to test molecules other than 3GC such as antibiotics (tetracycline and oxytetracycline), antiparasitics (ivermectin, flumethrin, fenbendazol, and amitraz), heavy metal [arsenic, HNO_3_, aluminum, HNO_3_, cadmium (CdSO_4_), zinc (ZnCl_2_), copper (CuSO_4_), iron (FeCl_3_), and aluminum (Al_2_SO_4_)], and antioxidant (butylated hydroxytoluene) as sources of selective pressure. Whole-genome sequencing using short read (Illumina™) and long read (Nanopore™) technologies was performed on 34 genomes. *In silico* gene screening and comparative analyses were used to characterize the genetic determinants of resistance, their mobility, and the genomic relatedness among isolates.

**Results:**

Our analysis unveiled a low diversity among the animal ESBL-producing strains. Notably, *E. coli* ST3268 was recurrently isolated from both flies (*n* = 9) and cattle (*n* = 5). These *E. coli* ST3268/*bla*_CTX-M-15_/*bla*_TEM-1B_ have accumulated multiple plasmids and genes, thereby representing a reservoir of resistance and virulence factors. Our findings suggest that flies could act as effective mechanical vectors for antimicrobial gene transfer and are capable of transporting resistant bacteria across different environments and to multiple hosts, facilitating the spread of pathogenic traits. A significantly higher mean minimum inhibitory concentration of oxytetracycline (841.4 ± 323.5 mg/L vs. 36.0 ± 52.6 mg/L, *p* = 0.0022) in ESBL *E. coli* than in non-ESBL *E. coli* and *bla*_CTX-M-15_ gene overexpression in oxytetracycline-treated vs. untreated ESBL *E. coli* (RQ_Oxy_ = 3.593, *p* = 0.024) confirmed oxytetracycline as a source of selective pressure in ESBL *E. coli*.

**Discussion:**

The occurrence of ESBL *E. coli* in a farm without 3GC use is probably due to an as yet undefined human origin of *Enterobacteriaceae bla*_CTX-M-15_ gene transmission to animals in close contact with cattle farm workers and the maintenance of the local ESBL *E. coli* reservoir by a high fly diversity and oxytetracycline selective pressure. These findings highlight the critical need for stringent vector control to mitigate antimicrobial resistance spread for preserving public health. Addressing this issue necessitates a multifaceted approach combining microbial genetics, vector ecology, and farm management practices.

## Graphical Abstract

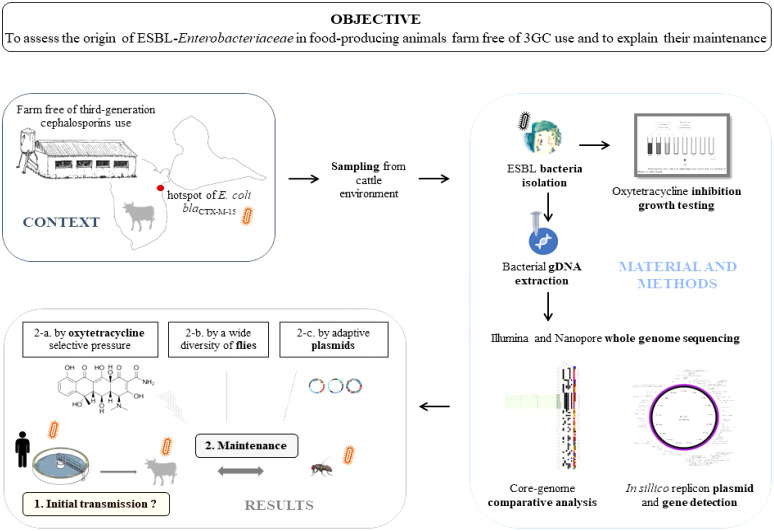

## 1 Introduction

Antimicrobial resistance (AMR) is currently one of the most important public health problems in the world (O’Neill, 2014). It has dramatically increased morbidity and mortality in both humans and animals (Eurosurveillance editorial team, 2015). The emergence of AMR is mainly due to the selective pressure of antibiotics used in both human and veterinary medicine (Nóbrega and Brocchi, 2014).

Food-producing animals are not only potential reservoirs of AMR but also central conduits through which resistance can be transmitted to humans. This transmission occurs *via* several vectors: the food chain (Antunes et al., 2020), direct contact, environmental contamination through waste (Heuer et al., 2011), and even indirectly through water sources (Juhna et al., 2007; Dierikx et al., 2013). Flies have been suggested to be involved in the dissemination of clones of antimicrobial-resistant bacteria and in the widespread dissemination of plasmids containing antimicrobial resistance genes between farms (Usui et al., 2015). Furthermore, it has been suggested that flies act as reservoirs of antimicrobial-resistant bacteria throughout their life cycle and may therefore be involved in their maintenance and circulation in the farm environment (Fukuda et al., 2019). However, the role of insects such as flies as vectors in the transmission of resistant bacteria within the complex ecosystem of a cattle farm has not been extensively studied. This gap is of particular importance given the ability of flies to bridge diverse ecological niches and move between animal waste, livestock, and human habitations, potentially serving as a critical conduit for pathogen spread. Studies have documented the transfer of extended-spectrum β-lactamase (ESBL) *E. coli* and genes from animals to farm workers, highlighting the complexity of these pathways (Dahms et al., 2015).

The threat posed by *Enterobacteriaceae* carrying ESBLs is alarming and global, with *E. coli* identified as the predominant species harboring ESBLs across in both humans (Ewers et al., 2012; Dahms et al., 2015) and animals (Dahms et al., 2015; Alonso et al., 2017). The presence of plasmids from distinct Inc groups (Michael et al., 2015) and phylogenetic lineages underscores the ability of these bacteria to spread efficiently and acquire resistance traits (Ewers et al., 2012; Lupo et al., 2018). In addition to antibiotics, other agents used in agriculture such as heavy metals and biocides (Wales and Davies, 2015) may also exert selective pressures that contribute to resistance.

Our study focuses on a cattle farm with a hotspot of ESBL *E. coli bla*_CTX-M-15_ carriers despite rational antimicrobial use and the absence of 3GC treatments (Gruel et al., 2021). Indeed the proportion of ESBL *E. coli* was significantly higher in this farm than in other farms (47.1% vs. 7,1%, *p* = 0.003). This result was difficult to explain. Furthermore, we demonstrated the role of animal food production systems as a reservoir of mobile genetic elements carrying multiple resistance determinants. However, the origin, spread, and maintenance of resistance were not established, and further studies are warranted to better define the genetic background of ESBL *E. coli* isolates and the context of antibiotic resistance in Guadeloupe, especially in food-producing animals not exposed to third-generation cephalosporins. Mechanisms other than the selective pressure of these antimicrobials in the emergence of antibiotic resistance remain to be elucidated. We investigate the hypothesis that other selective pressures, such as oxytetracycline and environmental factors, may play a role in the persistence of ESBL *Enterobacteriaceae*. Moreover, we explore the potential for human–animal transfer as a source of AMR. This work aims to elucidate the origins and maintenance mechanisms of AMR in cattle, potentially offering insights into mitigation strategies that address these resistance pathways at the ecosystem level.

## 2 Materials and methods

### 2.1 Sampling and collection

A total of 16 farms were visited and sampled between February 2018 and November 2019 (**Supplementary Data Set S1**). We focused our investigations on one farm, number 13, which had the highest rate of ESBL *E. coli* (Gruel et al., 2021). Between February 2018 and May 2019, 74 samples were collected only once at that farm. Fresh fecal samples were randomly collected from cattle living in the stall (*n* = 32) or in the field (*n* = 13) and from stalled goats (*n* = 10) immediately after defecation. We did not actually sample manure or goat feed. Flies that landed around cattle feces (*n* = 1), manure (*n* = 1), or goat breastfeeding food (pool *n* = 4) and adult mosquitoes in unused goat feeders (pool *n* = 1) were trapped using a 6-V mechanical aspirator. The mechanical aspiration technique used allowed the collection of pools of several flies: around cattle feces (*n* = 1) yielded 42 flies, manure (*n* = 1) yielded 81 flies, and goat breastfeeding food (*n* = 4) yielded 34 flies. A total of 157 flies were collected from six samples. Drinking water (*n* = 3) and untreated agricultural water (*n* = 2) were sampled. Wastewater samples (*n* = 3) were collected downstream of the administration building. Cattle feed (*n* = 1), solubilized goat milk (*n* = 1), milk powder (*n* = 1), and pellets (*n* = 1) were collected aseptically. All samples were stored and transported in sterile cups or bags on ice to the laboratory of the Institut Pasteur within 4 h.

### 2.2 Isolation and identification of bacteria

A 10-μL loop of each fecal sample was mixed in 10 mL of Luria–Bertani (LB) broth (BD Difco™, Humeau, La Chapelle-sur-Erdre, France). Suspensions of pellet, powdered milk, and food were prepared by mixing 30 g in 200 mL of LB. The flies and mosquitoes were crushed manually with a micropestle in 1 mL LB. A volume of 1 mL of wastewater sample was suspended in 10 mL of LB. The water (500 mL) was filtered through a 0.45-μm membrane (Millipore, Guyancourt, France), and the filter was incubated in 10 mL LB with 4 mg/L ceftriaxone for enrichment. The suspensions were supplemented with or without 4 mg/L ceftriaxone and incubated at 37°C for 24 h. Selective enrichments with 4 mg/L ceftriaxone were streaked onto chromogenic coliform agar plates (CHROMagar™, Paris, France) supplemented with 4 mg/L ceftriaxone. Non-selective enrichments were streaked onto chromogenic coliform agar plates without 4 mg/L ceftriaxone. All plates were incubated at 37°C for 24 h. Metallic blue colonies were randomly picked from the non-selective (*n* = 1) and selective (*n* = 4) chromogenic coliform agar, respectively. These isolates were then identified by matrix-assisted laser desorption/ionization time-of-flight mass spectrometry on an Axima high-performance spectrometer (Shimadzu Corp, Osaka, Japan). The susceptibility of all isolates to 17 antimicrobials in six different classes was assessed by the standard disk diffusion method on Mueller–Hinton agar, as previously described (Gruel et al., 2021).

### 2.3 Measurement of minimum inhibitory concentration

Minimum inhibitory concentration (MIC) values were used to compare the relative resistance levels of ESBL isolates with those of non-ESBL isolates. The MIC was determined using the EUCAST reference broth microdilution method (https://www.eucast.org/publications_and_documents/consultations/). Antibiotics (cefotaxime, ceftriaxone, tetracycline, and oxytetracycline), antiparasitics (ivermectin, flumethrin, fenbendazol, and amitraz), heavy metal [arsenic, HNO_3_, aluminum, HNO_3_, cadmium (CdSO_4_), zinc (ZnCl_2_), copper (CuSO_4_), iron (FeCl_3_), aluminum (Al_2_SO_4_)], and antioxidant (butylated hydroxytoluene) molecules were tested. Serial dilutions were inoculated with a pure bacterial suspension at 0.5 McFarland turbidity within 2 h of preparation. After overnight incubation at 37°C, the optical density at 620 nm (OD_620_) was measured using a microplate reader (Multiscan™ FC, Thermo Fisher Scientific). The MICs were read as the lowest concentrations that produced no visible growth. *E. coli* ATCC 25922 was used as the control strain. The listed MIC values presented are the mean of three independent experiments.

### 2.4 Molecular identification of flies

Flies (*n* = 157) from the sample pool were divided into eight groups based on their morphological characteristics. The taxonomic assignment of the fly species was performed on one fly from each of the eight morphotype groups. DNA was extracted individually from seven morphologically different flies using NucleoSpin® Tissue DNA Extraction Kit (Macherey-Nagel, Hoerdt, France) according to the manufacturer’s instructions. A fragment of the genes encoding cytochrome oxidase I (COI) (710 bp) was amplified in all flies as previously described (Folmer et al., 1994). Amplified PCR products were sequenced (Eurofins, Cologne, Germany) and compared to known COI gene sequences in the GenBank database by multiple sequence alignment using BLASTn (http://blast.ncbi.nlm.nih.gov/Blast.cgi). All matching sequences were submitted to the phylogenetic tree reconstruction pipelines available on the Phylogeny.fr platform (Dereeper et al., 2008). The tree was constructed using the “Advanced” option, which allows the statistical evaluation of branch support values using 100 bootstraps, and plotted using iTOL (Letunic and Bork, 2021) v6.7.4.

### 2.5 *bla*_CTX-M-15_ gene expression

To assess the selective advantage of ESBL *E. coli* under oxytetracycline, ivermectin, and copper selective pressure, *bla*_CTX-M-15_ gene expression was quantified and compared between treated and untreated isolates. The *bla*_CTX-M-15_ gene expression was determined in 14 ESBL *E. coli* isolates using a two-step RT-qPCR strategy described in detail in **Supplementary Material M1**. Briefly, bacterial samples were obtained from overnight-cultured ESBL and non-ESBL *E. coli* in Luria–Bertani broth media supplemented or not with oxytetracycline at a subinhibitory concentration. The bacterial density was measured by using a photometer and pelleted to adjust the concentration to 10^8^ cells/mL. Total RNA was extracted immediately using the NucleoSpin® RNA isolation kit following the manufacturer’s recommendations (Macherey-Nagel). A maximum of 2 µg of RNA was then reverse-transcribed to the corresponding cDNA using the SuperScript™ VILO™ Master Mix (Thermo Fisher Scientific), in a total volume of 20 µl, according to the manufacturer’s instructions. cDNA was then used in qPCR using the TaqMan™ Gene Expression Master Mix and thanks to a 7500 Real-Time PCR system (Thermo Fisher Scientific). 16S was the reference gene. For each run, a standard curve was generated in duplicate using a 10-fold serial dilution of a quantification calibrator of untreated *E. coli* cDNA. The 2^‐ΔΔCT^ algorithm was used to estimate the relative expression level of *bla*_CTX-M-15_ transcripts for the two populations studied using the RQ application module on the Thermo Fisher Cloud. Each real-time PCR run included the gene expression measurements of the endogenous 16S rRNA gene and the target *bla*_CTX-M-15_ gene in the corresponding samples.

### 2.6 Whole-genome and multiplex long read sequencing

A total of 34 genomes of *E. coli* isolates (*n* = 23) and *Enterobacter cloacae* complex Taxon 4 (*n* = 11) were obtained from farm number 13. To assess the genomic relatedness and dynamics of ESBL transmission, high-throughput whole-genome sequencing (WGS) of 79 isolates [34 ESBL *Escherichia coli* (*n* = 23) and *E. cloacae* complex Taxon 4 (*n* = 11) isolates from farm number 13 and 45 from other farms in Guadeloupe (Gruel et al., 2021)] was performed at the Biomics Platform, C2RT (Institut Pasteur, Paris, France). The preparation of the WGS libraries, the sequencing process, and the detailed analysis are described in **Supplementary Material M2**. Briefly, libraries were prepared using the Nextera XT kit (Illumina), and sequencing was performed on the NextSeq 500 system (Illumina), generating 35–151-bp paired-end reads for an average depth of coverage of 85-fold (minimum 78-fold, maximum 92-fold). The reads were trimmed and filtered. The genomes were assembled, and final quality was assessed. Annotation of the assembled genomes was performed, and then a core genome was extracted. Maximum likelihood phylogenetic reconstruction was performed and plotted on a tree. *In silico* screening and annotation of replicon plasmid types, antimicrobial resistance, virulence genes, and multilocus sequence typing (MLST) were performed. The same software tools were used to characterize plasmids (Chen et al., 2005; Wirth et al., 2006; Zankari et al., 2012; Carattoli and Hasman, 2020). The phylogenetic tree was constructed as described above. Genomic identification of *Enterobacter* strains was performed using the different approaches described in our previous manuscript (Pot et al., 2022). To fully reconstruct and characterize the major plasmids, 14 *bla*_CTX-M-15_ ESBL *E. coli* isolates were sequenced using Oxford Nanopore sequencing long-reads technology on a MinION device. The preparation of the MinION libraries, the sequencing procedure, and the detailed analysis are described in **Supplementary Material M3**. Briefly, libraries were constructed from 1 μg of unfragmented bacterial gDNA following the protocol instructions for native barcoded genomic DNA (using EXP-NBD104, EXP-NBD114, and SQK-LSK109). The final library was loaded onto a R9.4.1 flow cell (FLO-MIN106D) according to the manufacturer’s instructions and run on a laptop (MinKNOW Core v3.6.5). Single-flow cell sequencing data from multiplexed barcoded isolates were run on the MinION for 48 h. Base calling of MinION raw signals was performed. Fastq files were extracted and split by barcode. *De novo* genome assembly was performed using a hybrid strategy on combined nanopore long reads and previously available Illumina short reads. The fully resolved assemblies were generated and visualized. Quality control of nanopore data was performed. The plasmids were aligned graphically and annotated. Mobilization module characterization was performed.

## 3 Results

### 3.1 ESBL Enterobacteria carriage in wastewater, cattle, and fly species

**Figure 1:**
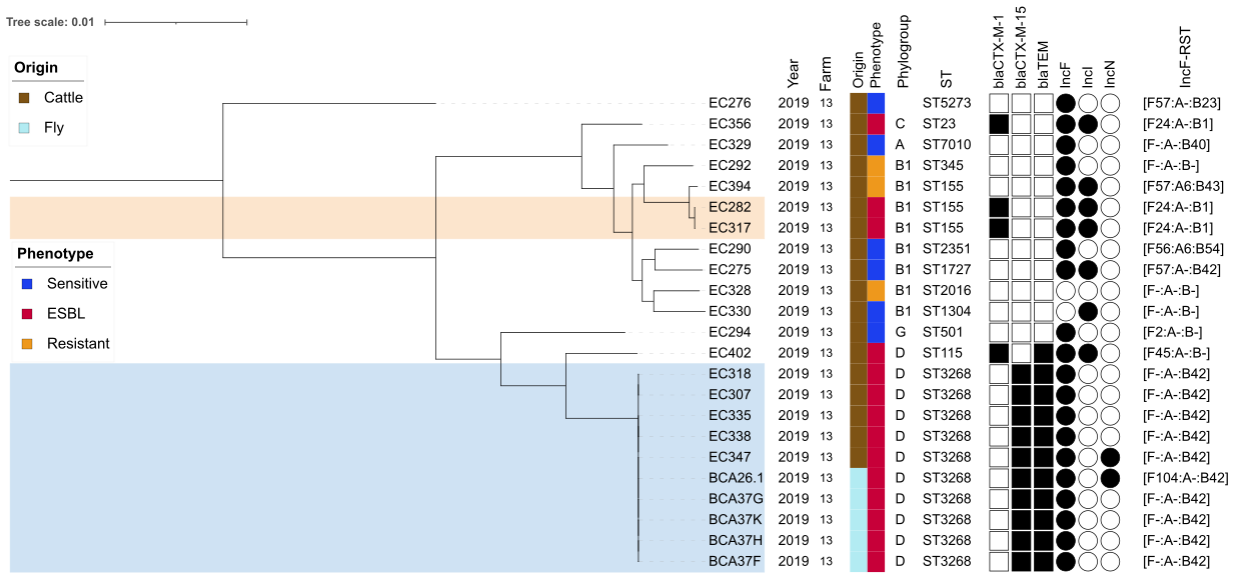
Core genome maximum likelihood phylogenetic tree of 23 *E. coli* isolates from farm number 13. Clusters ST3268 and ST155 represent groups of similar core genomes (0 to 25 single-nucleotide polymorphism difference). Hosts and antimicrobial susceptibility phenotypes are indicated by vertical colored stripes. The resistant phenotype is assigned to isolates that are resistant to at least one of the 17 antimicrobials tested. ST, sequenced type based on the Achtman MLST scheme (Wirth et al., 2006). Corresponding β-lactamase-associated resistance-coding genes are indicated by black squares and plasmids by black circles. IncF, plasmid incompatibility group F; RST, replicon typing system.

A total of 12 out of 74 samples (16.2%) were ESBL-positive. Of these, 25 ESBL Enterobacteria were isolated: 14 *E. coli* (**Table 1**, **Figure 1**) were isolated mainly from cattle in stalls and from five different fly species (**Supplementary Figure S1**) collected around goat breastfeeding food and manure. No ESBL isolates were detected elsewhere in the environmental samples from farm number 13. A total of 11 ESBL-producing Enterobacter isolated from wastewater downstream of the administration building were identified as belonging to *E. cloacae* complex Taxon 4 species according to the latest nomenclature (Feng et al., 2021). Their sequence type (ST) was ST598, and they differed from one to 23 single-nucleotide polymorphism (SNPs) (**Supplementary Figure S2**).

**Table 1:** Description of samples and ESBL enterobacteria collected from the farm environment.

Origin	Sample						Isolate		
*n* (%)	Total (*n* = 74)		ESBL + (*n* = 12)		Total^a^ (*n* = 34)		ESBL + (*n* = 25)		Taxonomy
Cattle feces									*Escherichia coli*
In stall	32	(43.2)	9	(12.2)	18	(52.9)	9	(26.5)	
In field	13	(17.6)	0	(0.0)	0	(0.0)	0	(0.0)	
Goat feces									*Escherichia coli*
In stabulation	10	(13.5)	0	(0.0)	0	(0.0)	0	(0.0)	
Flies									*Escherichia coli*
Around cattle feces	1	(1.4)	0	(0.0)	0	(0.0)	0	(0.0)	
Around manure	1	(1.4)	1	(1.4)	4	(11.8)	4	(11.8)	
Around goat breastfeeding food	4	(5.4)	1	(1.4)	1	(2.9)	1	(2.9)	
Mosquitoes									*Escherichia coli*
In goat feeder	1	(1.4)	0	(0.0)	0	(0.0)	0	(0.0)	
Water									*Escherichia coli*
Agricultural	2	(2.7)	0	(0.0)	0	(0.0)	0	(0.0)	
Drinking	3	(4.1)	0	(0.0)	0	(0.0)	0	(0.0)	
Milk									*Escherichia coli*
Solubilized	1	(1.4)	0	(0.0)	0	(0.0)	0	(0.0)	
Powder	1	(1.4)	0	(0.0)	0	(0.0)	0	(0.0)	
Food									*Escherichia coli*
Pellets	1	(1.4)	0	(0.0)	0	(0.0)	0	(0.0)	
Grass	1	(1.4)	0	(0.0)	0	(0.0)	0	(0.0)	
Wastewater									*Enterobacter cloacae*
Administration building	3	(4.1)	1	(1.4)	11	(32.4)	11	(32.4)	

ESBL, extended-spectrum β-lactamase producer; +, positive sample or isolate from corresponding sample.

a Isolates resistant to at least one of the following antibiotics: ampicillin, streptomycin, nalidixic acid, tetracycline, and trimethoprime-sulfamethoxazol.

### 3.2 A reservoir of *bla*_CTX-M-15_ ESBL isolates

A total of 25 ESBL Enterobacteriaceae genomes were sequenced from the 12 ESBL-positive samples, and nine additional genomes were provided from ESBL-negative samples. The 25 ESBL genomes from the 12 ESBL-positive samples were distributed as follows: five *E. coli* genomes from two pooled fly samples, nine *E. coli* genomes from nine cattle, and 11 *E. cloacae* genomes from one human wastewater sample. A total of 34 genomes of *E. coli* isolates (*n* = 23) and *E. cloacae* (*n* = 11) from farm number 13 were sequenced. Among the ESBL producers (*n* = 25), most of them carried the *bla*_CTX-M-15_ gene (21/25, 84.0%), followed by the *bla*_CTX-M-1_ gene (5/25, 20.0%; **Table 2**). Replicon genes from incompatible FIB group plasmids were found in all ESBL isolates from the three biotopes (25/25, 100.0%). However, there were differences between bacterial species (**Figure 1**). The IncFIB [F-:A-:B42] and IncFIB [F-:A-:B70] replicon sequence types were found in ESBL *E. coli* and ESBL *E. cloacae* complex Taxon 4, respectively. The IncN-pST3 replicon type was found only in cattle and fly ESBL *E. coli* ST3268.

**Table 2:** Distribution of *bla*_CTX-M_ gene and replicon type in extended-spectrum β-lactamase isolates.

	Total		*bla*_CTX-M-15_		*bla*_CTX-M-1_		IncFIB		IncN	
*n* (%)	(*n* = 25)		(*n* = 21)		(*n* = 4)		(*n* = 25)		(*n* = 2)	
Cattle	9	(36.0)	5	(20.0)	4	(16.0)	9	(36.0)	1	(4.0)
Fly	5	(20.0)	5	(20.0)	0	(0.0)	5	(20.0)	1	(4.0)
Wastewater	11	(44.0)	11	(44.0)	0	(0.0)	11	(44.0)	0	(0.0)

bla, β-lactamase.

### 3.3 An ecosystem with a high potential for resistance spread and persistence

**Figure 2:**
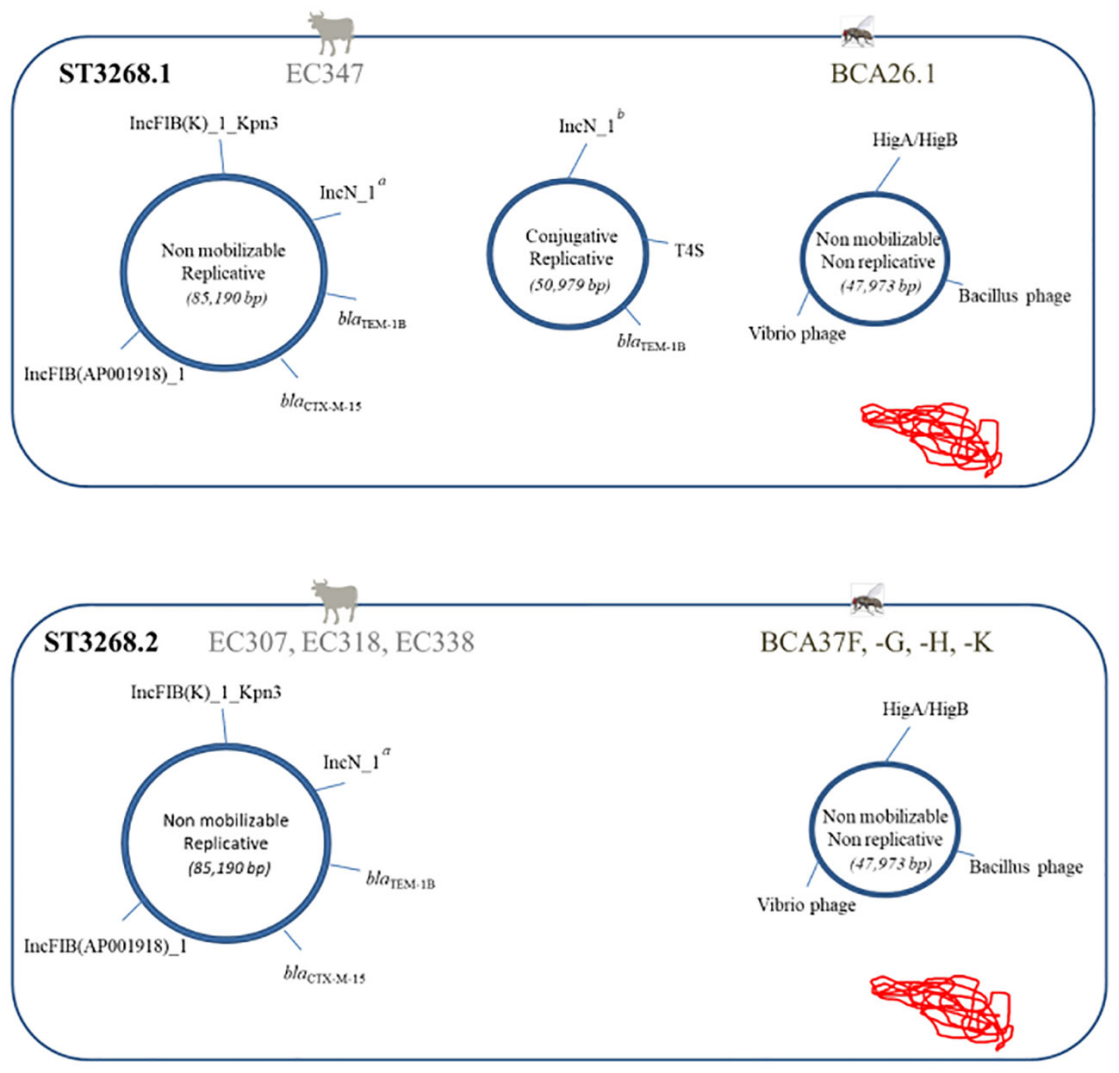
Schematic representation showing two combinations of the ultrastructural genetic background of the ST3268.1 ESBL *E. coli* subclusters (*n* = 7 isolates: BCA37F, -G, -H, -K, EC307, EC318, and EC338) and ST3268.2 (*n* = 2 isolates: BCA26.1 and EC347). Plasmids are shown as circles annotated for replicon, β-lactamase resistance genes, secretion system (T4S), toxin/antitoxin system (HigA/HigB), and phage-encoded protein genes. Supercoiled chromosomal DNA is schematically shown in red. a, truncated IncN_1_AY046276 replicon (247 bp). b, complete IncN_1_AY046276-pST3 replicon (512 bp).

Sequence assembly using long reads revealed that the *bla*_CTX-M-15_ gene of fly and bovine ESBL *E. coli* and wastewater ESBL *E. cloacae* was carried on the IncFIB [F-:A-:B42] and IncFIB [F-:A-:B70] replicon types, which differed in size and gene composition (**Supplementary Figure S3**). At the molecular level, plasmid reconstruction allowed the clustered ST3268 isolates to be divided into two new subclusters. Cluster ST3268.1 included the ST3268 isolates EC347 from cattle and BCA26.1 from flies, which simultaneously harbored three major plasmid backbones, and the ST3268.2 isolates (EC307, EC318, EC338 cattle and BCA37F, -G, -H, -K flies; **Figure 2**), which shared two plasmids with ST3268.1.

The *bla*_CTX-M-15_ gene was located in a transposon carried by a non-mobilizing multi-replicative plasmid IncFIB(K)_1_Kpn3_JN233704 (560 bp)/IncFIB(AP001918)_1_AP001918, cointegrated with a truncated IncN_1_AY046276 (85,190 bp), containing many mobile genetic elements (transposons, integrons, and insertion sequences) and several associated resistance genes. The conjugative replicon plasmid (IncN_1_AY046276-pST3, 50,979 bp), absent in ST3268.2, carrying the *bla*_TEM-1B_ gene with a cassette of resistance genes and virulence genes involved in the type IV secretion system (T4SS) was carried by ESBL *E. coli* strains common to cattle and flies. The third, a phage plasmid (47,973 bp), contained prophage regions from *Vibrio* and *Bacillus* without resistance genes and a toxin HigB/antitoxin HigA system involved in pathogenicity regulation. The EC335 isolates shared only the IncF 85,190-bp plasmid (not shown in **Figure 2**) with the other isolates from ST3268. ESBL *E. coli* ST3268-*bla*_CTX-M-15/TEM-1B_ was found here in cattle and flies (**Figures 1**, **2**). These results revealed an ESBL *E. coli* ST3268 cluster containing multiple plasmid backbones (**Supplementary Figure S3**), some of which are mobilizable with multiple associated resistance and virulence genes.

### 3.4 A first described IncF replicon [F-:A-:B42] in ST3268 ESBL *E. coli*

The collection of ST3268 isolates from other geographical origins found on Enterobase presents only ESBL producers (*n* = 22) (**Supplementary Figure S4**). Of these strains, 68.2% were isolated from humans (15/22). This sequence type was identified in many countries and was also found in wild and domestic animals with a *bla*_CTX-M-15_ gene. However, it has never been identified in insects, and the IncF replicon [F-:A-:B42] was only identified in farm number 13. No clonal relationship was found between the Guadeloupean isolates and those identified internationally.

### 3.5 Oxytetracycline selective pressure in favor of the emergence of ESBL *E. coli* IncN carriers

**Figure 3:**
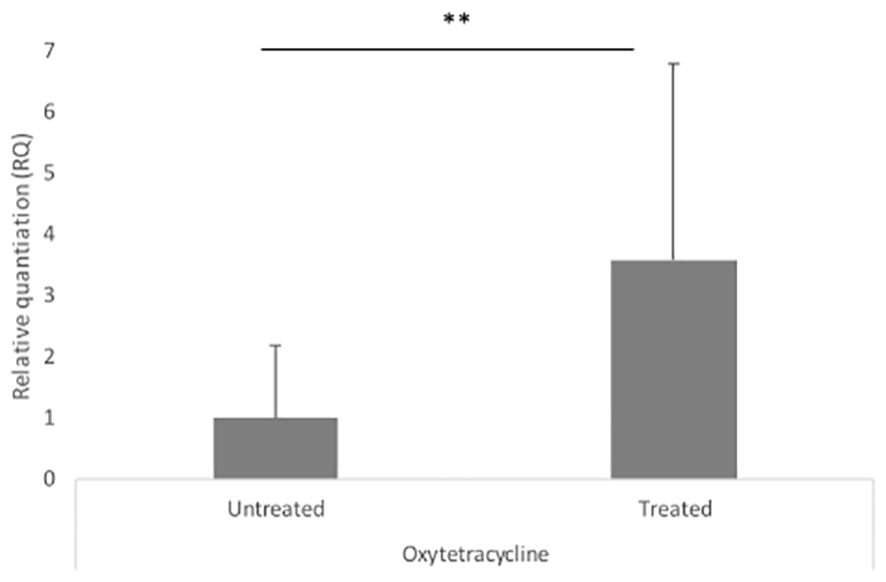
Modulation of *bla*_CTX-M-15_ gene expression under *in vitro* selective pressure. Relative quantification of *bla*_CTX-M-15_ gene expression under oxytetracycline treatment in ESBL *E. coli* isolates. Error bars represent the standard deviation of at least two independent experiments. RQ, relative quantification. Error bars indicate the range between RQ min and RQ max. ** Statistically significant p-value < 0.05.

We compared the MIC of 17 antiparasitic, antioxidant, antibiotic, and heavy metal compounds in ESBL (*n* = 14) *vs*. non-ESBL *E*. *coli* (*n* = 5) from farm number 13 (**Table 3**) and in ESBL *E. coli* full IncN carriers (ST3268.1, *n* = 3) *vs*. non-carriers (ST3268.2, *n* = 6). Our results showed a significantly higher mean MIC of oxytetracycline (841.4 ± 323.5 mg/L vs. 36.0 ± 52.6 mg/L, *p* = 0.0022) and arsenic (125.0 ± 0.0 mg/L vs. 78.1 ± 31.3 mg/L, *p* = 0.0019) in ESBL *E. coli* than in non-ESBL *E. coli*. Cefotaxime and ceftriaxone were used as 3GC-positive controls and confirmed a selective advantage of ESBL *E. coli*. Our results showed a higher tetracycline MIC (256 ± 0.0 mg/L vs. 170.7 ± 73.9 mg/L, *p* = 0.0325) in ESBL *E. coli* carrying the complete IncN conjugative T4SS replicon plasmid than in non-carriers. For arsenic, copper, and ivermectin, no difference in mean MIC was observed between ESBL *E. coli* complete IncN carriers (ST3268.1) and non-carriers (ST3268.2). In addition to MIC, our results showed a significant *bla*_CTX-M-15_ gene overexpression in oxytetracycline-treated vs. untreated ESBL *E. coli* (RQ_Oxy_=3.593, *p* = 0.024) (**Figure 3**). No difference in *bla*_CTX-M-15_ gene expression was observed with the ivermectin and copper treatments.

**Table 3:** Association of antibiotics, antiparasitics, heavy metals, and antioxidants with ESBL phenotype in *E. coli* isolates.

MIC (mg/L)	ESBL *E. coli*		Non-ESBL *E. coli*		*p-*value
	(*n* = 14)		(*n* = 5)		
	Mean	( ± SD)	Mean	( ± SD)	
Antimicrobials					
Cefotaxime	310.9	( ± 139.4)	51.6	( ± 114.3)	**0.0041**
Ceftriaxone	676.6	( ± 276.9)	205.4	( ± 457.6)	**0.0214**
Tetracycline	226.5	( ± 56.1)	20.4	( ± 27.6)	**0.0004**
Oxytetracycline	841.4	( ± 323.5)	36.0	( ± 52.6)	**0.0022**
Antiparasitics					
Ivermectin	512.0	( ± 0.0)	460.8	( ± 114.5)	0.0943
Flumethrin	438.9	( ± 211.3)	307.2	( ± 114.5)	0.1574
Fenbendazol	563.2	( ± 264.4)	512.0	( ± 443.4)	0.4673
Amitraz	384.0	( ± 132.8)	307.2	( ± 114.5)	0.2563
Heavy metals					
Arsenic (HNO_3_)	125.0	( ± 0.0)	78.1	( ± 31.3)	**0.0019**
Aluminum (HNO_3_)	200.0	( ± 64.5)	218.8	( ± 62.5)	0.6101
Cadmium (CdSO_4_)	151.8	( ± 53.2)	150.0	( ± 55.9)	0.9478
Zinc (ZnCl_2_)	571.4	( ± 181.6)	600.0	( ± 223.6)	0.7697
Copper (CuSO_4_)	2000.0	( ± 0.0)	2000.0	( ± 0.0)	–
Iron (FeCl_3_)	4285.7	( ± 1069.0)	4800.0	( ± 1,788.9)	0.4338
Aluminum (Al_2_SO_4_)	4000.0	( ± 0.0)	4000.0	( ± 0.0)	–
Antioxidant					
Butylated hydroxytoluene	329.1	( ± 120.0)	256.0	( ± 0.0)	0.1904

MIC, minimum inhibitory concentration; ESBL, extended-spectrum β-lactamase.

Mean ± SD or median IQR. Statistically significant p-values are highlighted in bold

### 3.6 Acquisition of distinct *E. coli* lineages

**Figure 4:**
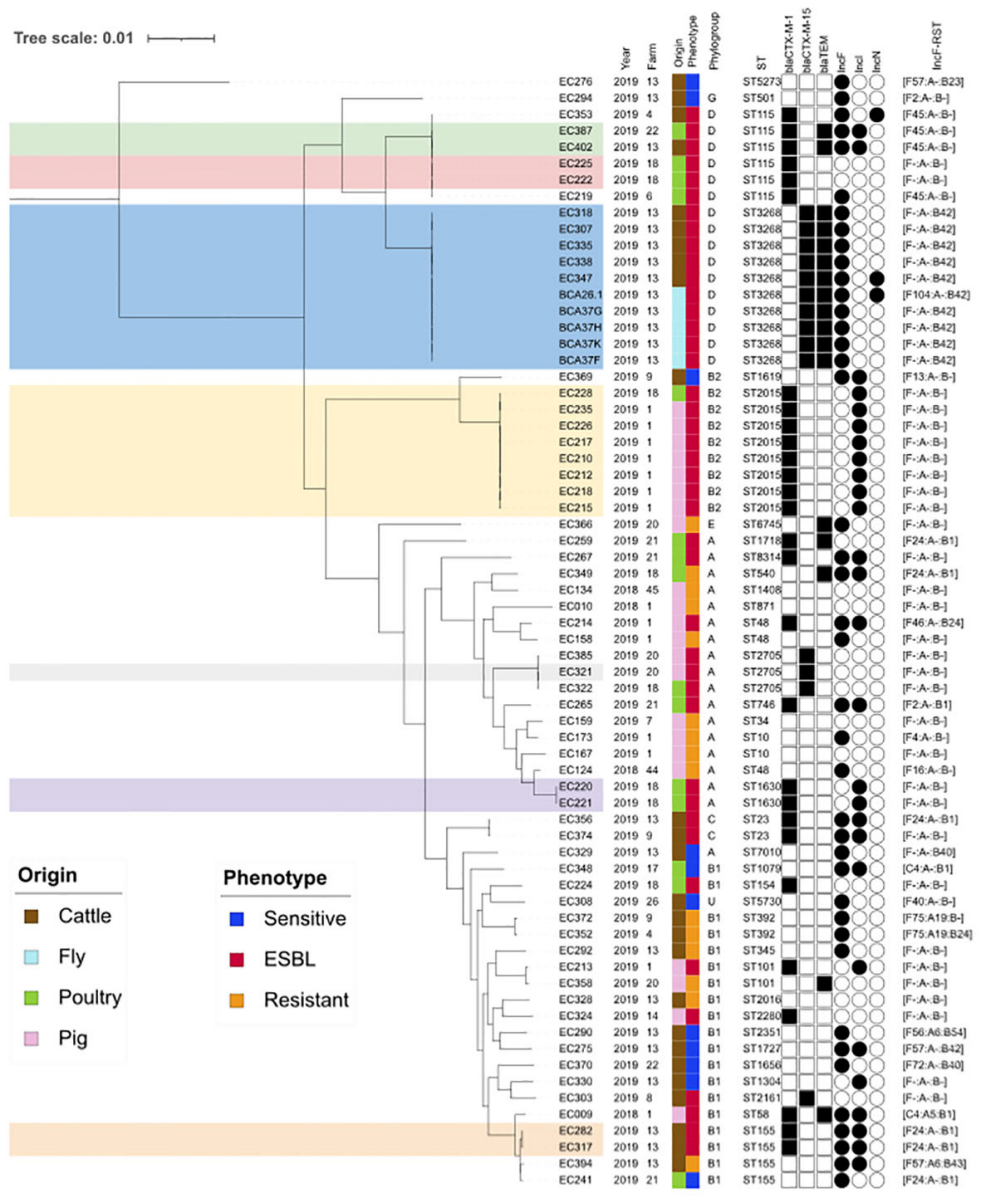
Core genome comparative analysis of 68 *E. coli* isolates from food-producing animals and flies. Maximum likelihood phylogenetic tree of 68 *E. coli* isolated from farms in Guadeloupe between 2018 and 2019. The farm number refers to our previous reference number (Gruel et al., 2021). Associated hosts and antimicrobial susceptibility phenotypes are indicated by vertical colored stripes. The resistant phenotype is assigned to isolates that are resistant to at least one of the 17 antimicrobials tested. Of these, 43 were ESBL producers. The colored clusters (ST115, ST3268, ST2015, ST2705, ST1630, and ST155) represent groups of similar core genomes (≤25 SNP). Only β-lactamase-encoding genes are indicated by black squares. Plasmid replicons are indicated by a black circle, and only the IncF RST was detailed. IncF, plasmid incompatibility group F; RST, replicon sequence typing.

To investigate the dynamics of *E. coli* circulation at a regional scale, we considered the 23 *E. coli* isolates from farm number 13 and 45 additional isolates from other farms surveyed in Guadeloupe during the same period. A total of 68 *E. coli* from 16 farms, including six cattle, six pig, and five poultry farms (one farm was a cattle and poultry producer), were included (**Figure 4**). A total of 29 isolates (42.6%) were grouped into seven clusters with similar core genomes (0 to 25 SNP difference). Four clusters representing (16/68, 23.5%) ESBL *E. coli* isolates were farm specific (10 ST3268 fly and cattle isolates from farm number 13, two ST115 poultry isolates from farm number 18, two ST1630 poultry isolates from farm number 18, and two ST155 cattle isolates from farm number 13), while three clusters (*n* = 13 ESBL isolates: ST2705, ST2015, and ST115) were from 11 different farms in the three food animal systems (**Figure 4**). The two largest clusters (ST3268 and ST2015) contained eight to 10 ESBL *E. coli* harboring a *bla*_CTX-M-1_ (ST2015) or a *bla*_CTX-M-15_ (ST3268) gene. Globally, the population structure of *E. coli* tends to show a higher proportion of unclustered isolates. When clustered, the isolates tend not to be farm specific. These results reflect sporadically acquired isolates from different lineages rather than the active spread of major clones. Cluster ST3268 showed a close genomic relationship between 10 CTX-M-15 producing *E. coli* from both fly and cattle sources. These *E. coli* ST3268/*bla*_CTX-M-15_/*bla*_TEM-1B_ have accumulated and maintained multiple plasmids and genes, thereby representing an extensive reservoir of resistance and virulence factors. Our results suggest that flies could act as vectors and highlight a clear link between cattle and flies in the spread of CTX-M-15 producing *E. coli.* This underscores the role of flies in increasing the risk of transmission of such resistance factors from livestock to the wider environment. This refined statement underscores the importance of understanding these dynamics in addressing the spread of antibiotic resistance.

## 4 Discussion

This study investigates the origin of ESBL *E. coli* in a farm of food-producing animals not exposed to third-generation cephalosporins, allowing the identification of a local cattle and fly reservoir of *E. coli* ST3268/*bla*_CTX-M-15_/*bla*_TEM-1B_. This ST was not found elsewhere in Guadeloupe (Sadikalay et al., 2018; Guyomard-Rabenirina et al., 2020; Gruel et al., 2021, 2022). This ST was also rarely found in genomic databases. However, it has been recovered from different compartments worldwide (Zhou et al., 2020). Its association with the *bla*_CTX-M-15_ gene was first identified in humans in France in 2010 (Zamudio et al., 2022). ST3268 ESBL *E. coli* was subsequently described in cattle (Hordijk et al., 2019) and in raccoons (Zhou et al., 2020). Although the reservoirs (flies and cattle) of *E. coli* ST3268/*bla*_CTX-M-15_/*bla*_TEM-1B_ are limited to one farm and the human compartment still seems to be sporadically affected by this ST, special caution is required as we are facing a new reservoir of an emerging zoonotic *E. coli* ST3268 lineage (Hammerum et al., 2020). The emergence of a novel *E. coli* lineage, ST3268, harboring resistance genes common to both cattle and flies is significant. It suggests that vectors such as flies may play a role in the maintenance and spread of novel and important resistance genes, with potential implications for both animal and human health. This reinforces the need for integrated veterinary and public health surveillance and control strategies. To reduce the risk of flies as vectors, we advocate improved farm hygiene and waste management practices, the use of biosecurity measures such as insect screens and zappers, and further research into environmentally friendly insect control methods.

In our study, *E. coli* ST3268/*bla*_CTX-M-15_/*bla*_TEM-1B_ isolated from flies and cattle have accumulated multiple plasmids and genes and represent a reservoir of resistance and virulence factors. In all ESBL *E. coli* isolates, the *bla*_CTX-M-15_ gene was carried by a non-mobile multireplicon plasmid (2 IncFIB) cointegrating with a truncated IncN_1_AY046276 replicon. ESBL *E. coli bla*_CTX-M-15_/*bla*_TEM-1B_ of the ST38 clonal group has already been found in Japan on unsequenced but transferable IncFIB plasmids (Usui et al., 2013) shared between cattle and flies. However, to the best of our knowledge, our multidrug resistance structure of IncFIB/*bla*_CTX-M-15/_ *bla*_TEM1B_ multi-FIB replicon cointegrating IncN has never been described in animal ESBL *E. coli*. The conjugative plasmid IncN-pST3 found in flies and cattle is enriched in resistance and virulence genes that can spread to humans and cause severe infections that are difficult to treat with current antibiotics. Since the IncF/*bla*_CTX-M-15_ non-mobilized plasmid backbone differed between animal [IncFIB (F-:A-:B42), 85,190 bp] and wastewater [IncFIB (F-:A-:B70), 106,354 bp], the origin of the human *bla*_CTX-M-15_ genes observed in flies and cattle is a consequence of multiple and cumulative origins of ESBL bacteria rather than the active horizontal spread of a single successful clone or plasmid. We investigated here the main sources of ESBL *E. coli* originated from animals, insects, water, feeds, and human wastewater. The ESBL-producing *E. cloacae* Taxon 4 ST598 found in administrative building wastewater was previously found in hospital wastewater in Guadeloupe and also isolated from patients (Pot et al., 2022). These findings highlight the importance of investigating non-animal or non-human reservoirs of antibiotic-resistant bacteria, as they may play a key role in the spread of resistance and may reach humans through various transmission routes. Other possible sources of ESBL *E. coli* include incoming animals, soil (Gelalcha et al., 2022), or wild fauna (Guyomard-Rabenirina et al., 2020) not investigated here. As no ESBL *E. coli* were detected in cattle in the field or in the grass and no manure was applied on soil, our hypotheses did not support a soil source of ESBL *E. coli* (Collis et al., 2022). Incoming animals are not involved in our agroecosystem. Thus, the most alternative source of ESBL *E. coli* on farm number 13 may be from wild fauna, particularly rats, which are very present in farm housing and have already been described as carriers of ESBL *E. coli* (Guyomard-Rabenirina et al., 2020). Due to the hygienic measures taken after our visit, no rat feces were found at farm number 13.

In our study, several ESBL *E. coli* isolates combined broad and narrow host range plasmids (Rozwandowicz et al., 2018). In addition, the plasmids in each group contain different combinations of resistance and virulence genes. Taken together, these results may explain the successful persistence and spread of ESBL isolates and plasmids on the farm and suggest a complex transmission dynamics of resistance and virulence genes, plasmids, and *Enterobacteriaceae* strains. This reflects the spread of multiple persistent ESBL isolate lineages rather than a single epidemic circulating clone. *E. coli* ST3268 was found to host multiple plasmids carried by different fly species. Due to their strong flight capabilities (Nazni et al., 2005), flies appear to be the primary vector for the spread of ESBL isolates in our ecosystem and could act to spread resistance genes (Usui et al., 2015). The current dogma dictates that antimicrobial resistance is associated with a fitness cost. The fitness cost of plasmids in our Enterobacteriaceae has not been evaluated, but we are likely facing contemporary ESBL *E. coli* strains that may be more “fit” and able to persist in the gut with a significant colonization burden despite a lack of antibiotic exposure (Kremer et al., 2022). Indeed some plasmids have evolved to have little effect on host strains (Cottell et al., 2012). Therefore, the persistence of antibiotic resistance genes and their vectors can be expected in the absence of antibiotic selection pressure, regardless of antibiotic stewardship. Other means of reducing plasmid stability are needed to prevent the persistence of these vectors and the antibiotic resistance genes that they carry. Differences in plasmid characteristics between samples highlight the complexity of transmission dynamics. Our study contributes to the understanding of how resistance genes spread, with implications for approaches to monitoring and controlling AMR on farms, and the importance of considering a variety of genetic vehicles in these processes. The coexistence of multiple resistant and mobilizable plasmids has serious implications for both the agricultural ecosystem and public health. It demonstrates the ability of pathogens to evolve rapidly in response to environmental pressures and the need for comprehensive genomic surveillance strategies to monitor and understand this genetic exchange.

The isolation of *E. coli* resistant to the 3GC cefotaxime from cattle with no previous exposure to cefotaxime has recently been reported (Udikovic-Kolic et al., 2014; Mir et al., 2016; Liu et al., 2020). In our study, the occurrence of ESBL isolates in cattle was probably due to the co-selection of multiple resistance genes in the same plasmid by other antibiotics such as oxytetracycline. A metagenomic study of bacterial communities showed that tetracycline resistance is frequently found and transmitted with ESBL-containing plasmids (Tacão et al., 2014). The widespread use of tetracyclines in Guadeloupe (Gruel et al., 2021) may explain some of the discrepancy between the high prevalence of resistance and the moderate use of 3GC. Tetracyclines that are not listed as critical for human treatment may promote resistance to more important molecules. Our results show a selective advantage of ESBL *E. coli* carrying IncN_1_AY046276 over non-carriers under oxytetracycline selective pressure, but this needs to be strengthened by more consistent sampling to increase the robustness of the assays and confirm the trends. These results confirm previous conclusions that the maintenance of plasmids in bacteria, and thus the *bla*_CTX-M_ genes, is a contribution of genetic determinants mediating non-β-lactam resistance mechanisms acquired through co-selection (Woodford et al., 2009). In addition, the presence of ESBL *E. coli* in a 3GC-free environment suggests that alternative selective pressures may be at play. It highlights the possibility of other contributing factors, such as the use of different antimicrobials such as oxytetracycline, heavy metal exposure, and non-antibiotic selective agents, which could co-select for resistance. Our work calls for a re-evaluation of the current understanding of AMR transmission and highlights the need to consider a wider range of selective pressures. The identification of oxytetracycline as a potential selective agent for ESBL-producing bacteria highlights the need for comprehensive stewardship that includes all antibiotics, not just those thought to directly select for resistance. It contributes to a more nuanced approach to antibiotic use in agriculture.

While the design of this study primarily focused on investigating the role of the human compartment through the analysis of wastewater, we acknowledge the opportunity to extend our research by exploring ESBL *Enterobacteriaceae* presence among farm workers. Such an extension would not only complement our current findings but also offer a more comprehensive understanding of contamination origins, thereby enhancing the robustness of proposed risk mitigation strategies. Despite this, the integrity and relevance of the results presented remain intact. Future investigations, including longitudinal monitoring of strains among farm workers, are indeed recommended to fill this gap, further strengthening the study’s impact on preventing the emergence and spread of ESBL clones in such environments.

## 5 Conclusion

We demonstrated that the high level of ESBL *E. coli* in a farm without 3GC use is likely due to the maintenance of the local ESBL *E. coli* animal reservoir by a high fly diversity and oxytetracycline pressure. This is the first observation of multiple *E. coli* IncFIB/IncN::*bla*_CTX-M-15_/*bla*_TEM-1B_ replicon plasmids clustering in animals. While the likely human origin of this plasmid observed in flies and cattle remains to be clarified, our study highlights the importance of considering environmental factors and antibiotic stewardship in managing antimicrobial resistance. It shows that multiple factors, including the use of specific antibiotics, contribute to the selection of resistance genes, requiring a comprehensive strategy that includes monitoring drug use, regulating potential environmental contributors to AMR, and implementing biosecurity to reduce vector spread. These findings call for a One Health approach that integrates human, animal, and environmental health to inform policy and improve agricultural practices.

## Data availability statement

The datasets presented in this study can be found in online repositories. The names of the repository/repositories and accession number(s) can be found below: BioProject, PRJNA1001660.

## Ethics statement

As fecal samples were taken from animals in the field after excretion, “use of live animals for scientific purposes” (within the meaning of the Rural Code, Art R214-87 and following) was not relevant; no invasive procedure was conducted on live animals. Furthermore, the project was considered to be outside the scope of the regulations on animal experimentation by the chairman of the Ethics Committee on animal experimentation of the Antilles and Guyane (registered with the French Ministry of Higher Education, Research and Innovation No. 69). Thus, according to French national law for the protection of animals (No. 2013–118), which reproduces European directive 2010/63/UE on the protection of animals used for experimental and other scientific purposes, no ethics committee approval was deemed necessary according to Article 7.1 on recommendations for animal welfare and Article 7.8 on use of animals in research and education of the World Organization for Animal Health Terrestrial Animal Health Code, used in France. The entity responsible for the animals was the owners. Sampling of animals feces was authorized verbally by the owners, who are responsible for the animals. The studies were conducted in accordance with the local legislation and institutional requirements. Written informed consent was obtained from the owners for the participation of their animals in this study.

## Author contributions

AC: Data curation, Formal Analysis, Investigation, Writing – review & editing, Validation. DC: Writing – review & editing, Data curation, Formal Analysis, Software, Validation, Resources. GG: Data curation, Writing – review & editing, Investigation. IQ: Data curation, Writing – review & editing, Formal Analysis, Software, Visualization. MP: Formal Analysis, Visualization, Writing – review & editing, Validation, Investigation. RA: Writing – review & editing, Investigation, Resources. AD: Resources, Writing – review & editing, Software, Validation, Data curation, Formal Analysis. J-CB: Writing – review & editing, Investigation, Methodology. AT: Writing – review & editing, Funding acquisition. SF: Project administration, Validation, Writing – review & editing, Conceptualization, Data curation, Formal Analysis, Investigation, Methodology, Supervision, Writing – original draft, Visualization.

## References

[B1] Alonso C. A. Zarazaga M. Ben Sallem R. Jouini A. Ben Slama K. Torres C. (2017). Antibiotic resistance in *Escherichia coli* in husbandry animals: the African perspective. Lett. Appl. Microbiol. 64, 318–334. doi: 10.1111/lam.12724 28208218

[B2] Antunes P. Novais C. Peixe L. (2020). Food-to-humans bacterial transmission. Microbiol. Spectr 8, 2–26. doi: 10.1128/microbiolspec.MTBP-0019-2016 PMC10810214 31950894

[B3] Carattoli A. Hasman H. (2020). PlasmidFinder and in silico pMLST: identification and typing of plasmid replicons in whole-genome sequencing (WGS). Methods Mol. Biol. 2075, 285–294. doi: 10.1007/978-1-4939-9877-7_20 31584170

[B4] Chen L. Yang J. Yu J. Yao Z. Sun L. Shen Y.. (2005). VFDB: a reference database for bacterial virulence factors. Nucleic Acids Res. 33, D325–D328. doi: 10.1093/nar/gki008 15608208 PMC539962

[B5] Collis R. M. Biggs P. J. Burgess S. A. Midwinter A. C. Brightwell G. Cookson A. L. (2022). Prevalence and distribution of extended-spectrum β-lactamase and AmpC-producing *Escherichia coli* in two New Zealand dairy farm environments. Front. Microbiol. 13. doi: 10.3389/fmicb.2022.960748 PMC9403332 36033848

[B6] Cottell J. L. Webber M. A. Piddock L. J. V. (2012). Persistence of transferable extended-spectrum-β-lactamase resistance in the absence of antibiotic pressure. Antimicrob. Agents Chemother. 56, 4703–4706. doi: 10.1128/AAC.00848-12 22710119 PMC3421869

[B7] Dahms C. Hübner N.-O. Kossow A. Mellmann A. Dittmann K. Kramer A. (2015). Occurrence of ESBL-producing *escherichia coli* in livestock and farm workers in mecklenburg-western pomerania, Germany. PloS One 10, 1–13. doi: 10.1371/journal.pone.0143326 PMC4659621 26606146

[B8] Dereeper A. Guignon V. Blanc G. Audic S. Buffet S. Chevenet F.. (2008). Phylogeny.fr: robust phylogenetic analysis for the non-specialist. Nucleic Acids Res. 36, W465–W469. doi: 10.1093/nar/gkn180 18424797 PMC2447785

[B9] Dierikx C. M. van der Goot J. A. Smith H. E. Kant A. Mevius D. J. (2013). Presence of ESBL/ampC -producing *escherichia coli* in the broiler production pyramid: A descriptive study. PloS One 8, e79005. doi: 10.1371/journal.pone.0079005 24244401 PMC3820706

[B10] Eurosurveillance editorial team (2015). WHO member states adopt global action plan on antimicrobial resistance. Euro Surveill 20, 1. doi: 10.2807/ese.20.21.21137-en 26062562

[B11] Ewers C. Bethe A. Semmler T. Guenther S. Wieler L. H. (2012). Extended-spectrum β-lactamase-producing and AmpC-producing *Escherichia coli* from livestock and companion animals, and their putative impact on public health: a global perspective. Clin. Microbiol. Infect. 18, 646–655. doi: 10.1111/j.1469-0691.2012.03850.x 22519858

[B12] Feng Y. Hu Y. Zong Z. (2021). Reexamining the association of ampC variants with enterobacter species in the context of updated taxonomy. Antimicrob. Agents Chemother. 65, e0159621. doi: 10.1128/AAC.01596-21 34516244 PMC8597755

[B13] Folmer O. Black M. Hoeh W. Lutz R. Vrijenhoek R. (1994). DNA primers for amplification of mitochondrial cytochrome c oxidase subunit I from diverse metazoan invertebrates. Mol. Mar. Biol. Biotechnol. 3, 294–299. 7881515

[B14] Fukuda A. Usui M. Okamura M. Dong-Liang H. Tamura Y. (2019). Role of flies in the maintenance of antimicrobial resistance in farm environments. Microb. Drug Resist. 25, 127–132. doi: 10.1089/mdr.2017.0371 29708845

[B15] Gelalcha B. D. Ensermu D. B. Agga G. E. Vancuren M. Gillespie B. E. D’Souza D. H.. (2022). Prevalence of antimicrobial resistant and extended-spectrum beta-lactamase-producing *escherichia coli* in dairy cattle farms in east tennessee. Foodborne Pathog. Dis. 19, 408–416. doi: 10.1089/fpd.2021.0101 35451874

[B16] Gruel G. Couvin D. Guyomard-Rabenirina S. Arlet G. Bambou J.-C. Pot M.. (2022). High Prevalence of *bla* (CTXM-1)/IncI1-Iγ/ST3 Plasmids in Extended-Spectrum β-Lactamase-Producing *Escherichia coli* Isolates Collected From Domestic Animals in Guadeloupe (French West Indies). Front. Microbiol. 13. doi: 10.3389/fmicb.2022.882422 PMC9149308 35651489

[B17] Gruel G. Sellin A. Riveiro H. Pot M. Breurec S. Guyomard-Rabenirina S.. (2021). Antimicrobial use and resistance in *Escherichia coli* from healthy food-producing animals in Guadeloupe. BMC Vet. Res. 17, 116. doi: 10.1186/s12917-021-02810-3 33685450 PMC7938459

[B18] Guyomard-Rabenirina S. Reynaud Y. Pot M. Albina E. Couvin D. Ducat C.. (2020). Antimicrobial resistance in wildlife in Guadeloupe (French west indies): distribution of a single blaCTX–M–1/incI1/ST3 plasmid among humans and wild animals. Front. Microbiol. 11. doi: 10.3389/fmicb.2020.01524 PMC7366356 32754130

[B19] Hammerum A. M. Porsbo L. J. Hansen F. Roer L. Kaya H. Henius A.. (2020). Surveillance of OXA-244-producing *Escherichia coli* and epidemiologic investigation of cases, Denmark, January 2016 to August 2019. Eurosurveillance 25, 1–9. doi: 10.2807/1560-7917.ES.2020.25.18.1900742 PMC7219033 32400363

[B20] Heuer H. Schmitt H. Smalla K. (2011). Antibiotic resistance gene spread due to manure application on agricultural fields. Curr. Opin. Microbiol. 14, 236–243. doi: 10.1016/j.mib.2011.04.009 21546307

[B21] Hordijk J. Fischer E. A. J. van Werven T. Sietsma S. Van Gompel L. Timmerman A. J.. (2019). Dynamics of faecal shedding of ESBL- or AmpC-producing *Escherichia coli* on dairy farms. J. Antimicrob. Chemother. 74, 1531–1538. doi: 10.1093/jac/dkz035 30753489 PMC6524482

[B22] Juhna T. Birzniece D. Larsson S. Zulenkovs D. Sharipo A. Azevedo N. F.. (2007). Detection of *Escherichia coli* in biofilms from pipe samples and coupons in drinking water distribution networks. Appl. Environ. Microbiol. 73, 7456–7464. doi: 10.1128/AEM.00845-07 17720845 PMC2168204

[B23] Kremer A. Whitmer G. Diaz A. Sajwani A. Navarro A. Arshad M. (2022). ESBL *escherichia coli* isolates have enhanced gut colonization capacity compared to non-ESBL strains in neonatal mice. Microbiol. Spectr 10, e0058222. doi: 10.1128/spectrum.00582-22 36121240 PMC9603109

[B24] Letunic I. Bork P. (2021). Interactive Tree Of Life (iTOL) v5: an online tool for phylogenetic tree display and annotation. Nucleic Acids Res. 49, W293–W296. doi: 10.1093/nar/gkab301 33885785 PMC8265157

[B25] Liu Y. Dyall-Smith M. Marenda M. Hu H.-W. Browning G. Billman-Jacobe H. (2020). Antibiotic resistance genes in antibiotic-free chicken farms. Antibiotics 9, 1–12. doi: 10.3390/antibiotics9030120 PMC7148458 32183177

[B26] Lupo A. Saras E. Madec J. Y. Haenni M. (2018). Emergence of *bla*CTX-M-55 associated with *fos*A, *rmt*B and *mcr* gene variants in *Escherichia coli* from various animal species in France. J. Antimicrob. Chemother. 73, 867–872. doi: 10.1093/jac/dkx489 29340602

[B27] Michael G. B. Freitag C. Wendlandt S. Eidam C. Feßler A. T. Lopes G. V.. (2015). Emerging issues in antimicrobial resistance of bacteria from food-producing animals. Future Microbiol. 10, 427–443. doi: 10.2217/fmb.14.93 25812464

[B28] Mir R. A. Weppelmann T. A. Johnson J. A. Archer D. Morris J. G. J. Jeong K. C. (2016). Identification and characterization of cefotaxime resistant bacteria in beef cattle. PloS One 11, e0163279. doi: 10.1371/journal.pone.0163279 27642751 PMC5028047

[B29] Nazni W. A. Luke H. Wan Rozita W. M. Abdullah A. G. Sa’diyah I. Azahari A. H.. (2005). Determination of the flight range and dispersal of the house fly, *Musca domestica* (L.) using mark release recapture technique. Trop. Biomed. 22, 53–61. 16880754

[B30] Nóbrega D. B. Brocchi M. (2014). An overview of extended-spectrum beta-lactamases in veterinary medicine and their public health consequences. J. Infect. Dev. Ctries 8, 954–960. doi: 10.3855/jidc.4704 25116659

[B31] O’Neill J. (2014). Antimicrobial Resistance: Tackling a crisis for the health and wealth of nations. Rev. Antimicrob. Resist. 1–20. Available at: https://wellcomecollection.org/works/rdpck35v.

[B32] Pot M. Reynaud Y. Couvin D. Dereeper A. Ferdinand S. Bastian S.. (2022). Emergence of a Novel Lineage and Wide Spread of a *bla*(CTX-M-15)/IncHI2/ST1 Plasmid among Nosocomial Enterobacter in Guadeloupe. Antibiot (Basel Switzerland) 11, 1–20. doi: 10.3390/antibiotics11101443 PMC9598596 36290101

[B33] Rozwandowicz M. Brouwer M. S. M. Fischer J. Wagenaar J. A. Gonzalez-Zorn B. Guerra B.. (2018). Plasmids carrying antimicrobial resistance genes in *Enterobacteriaceae* . J. Antimicrob. Chemother. 73, 1121–1137. doi: 10.1093/jac/dkx488 29370371

[B34] Sadikalay S. Reynaud Y. Guyomard-Rabenirina S. Falord M. Ducat C. Fabre L.. (2018). High genetic diversity of extended-spectrum β-lactamases producing *Escherichia coli* in feces of horses. Vet. Microbiol. 219, 117–122. doi: 10.1016/j.vetmic.2018.04.016 29778183

[B35] Tacão M. Moura A. Correia A. Henriques I. (2014). Co-resistance to different classes of antibiotics among ESBL-producers from aquatic systems. Water Res. 48, 100–107. doi: 10.1016/j.watres.2013.09.021 24091187

[B36] Udikovic-Kolic N. Wichmann F. Broderick N. A. Handelsman J. (2014). Bloom of resident antibiotic-resistant bacteria in soil following manure fertilization. Proc. Natl. Acad. Sci. U S A 111, 15202–15207. doi: 10.1073/pnas.1409836111 25288759 PMC4210343

[B37] Usui M. Iwasa T. Fukuda A. Sato T. Okubo T. Tamura Y. (2013). The role of flies in spreading the extended-spectrum β-lactamase gene from cattle. Microb. Drug Resist. 19, 415–420. doi: 10.1089/mdr.2012.0251 23659602

[B38] Usui M. Shirakawa T. Fukuda A. Tamura Y. (2015). The role of flies in disseminating plasmids with antimicrobial-resistance genes between farms. Microb. Drug Resist. 21, 562–569. doi: 10.1089/mdr.2015.0033 26061440

[B39] Wales A. D. Davies R. H. (2015). Co-selection of resistance to antibiotics, biocides and heavy metals, and its relevance to foodborne pathogens. Antibiotics 4, 567–604. doi: 10.3390/antibiotics4040567 27025641 PMC4790313

[B40] Wirth T. Falush D. Lan R. Colles F. Mensa P. Wieler L. H.. (2006). Sex and virulence in *Escherichia coli*: an evolutionary perspective. Mol. Microbiol. 60, 1136–1151. doi: 10.1111/j.1365-2958.2006.05172.x 16689791 PMC1557465

[B41] Woodford N. Carattoli A. Karisik E. Underwood A. Ellington M. J. Livermore D. M. (2009). Complete nucleotide sequences of plasmids pEK204, pEK499, and pEK516, encoding CTX-M enzymes in three major *Escherichia coli* lineages from the United Kingdom, all belonging to the international O25:H4-ST131 clone. Antimicrob. Agents Chemother. 53, 4472–4482. doi: 10.1128/AAC.00688-09 19687243 PMC2764225

[B42] Zamudio R. Boerlin P. Beyrouthy R. Madec J.-Y. Schwarz S. Mulvey M. R.. (2022). Dynamics of extended-spectrum cephalosporin resistance genes in *Escherichia coli* from Europe and North America. Nat. Commun. 13, 7490. doi: 10.1038/s41467-022-34970-7 36509735 PMC9744880

[B43] Zankari E. Hasman H. Cosentino S. Vestergaard M. Rasmussen S. Lund O.. (2012). Identification of acquired antimicrobial resistance genes. J. Antimicrob. Chemother. 67, 2640–2644. doi: 10.1093/jac/dks261 22782487 PMC3468078

[B44] Zhou Z. Alikhan N.-F. Mohamed K. Fan Y. Achtman M. (2020). The EnteroBase user’s guide, with case studies on Salmonella transmissions, *Yersinia pestis* phylogeny, and Escherichia core genomic diversity. Genome Res. 30, 138–152. doi: 10.1101/gr.251678.119 31809257 PMC6961584

