# Supplementary material for: The fly route of extended-spectrum-β-lactamase-producing Enterobacteriaceae dissemination in a cattle farm: from the ecosystem to the molecular scale
Source: Front Antibiot. 2024 Apr 10;3:1367936. doi: 10.3389/frabi.2024.1367936 (PMC11732033; doi:10.3389/frabi.2024.1367936)
Supplement: Supplementary file 1 [file DataSheet_1.zip › Supplementary Figure S1.DOCX]

**Supplementary Figure S1.** Genetic relatedness of fly species based on cytochrome oxidase I (COI) gene fragment sequencing.


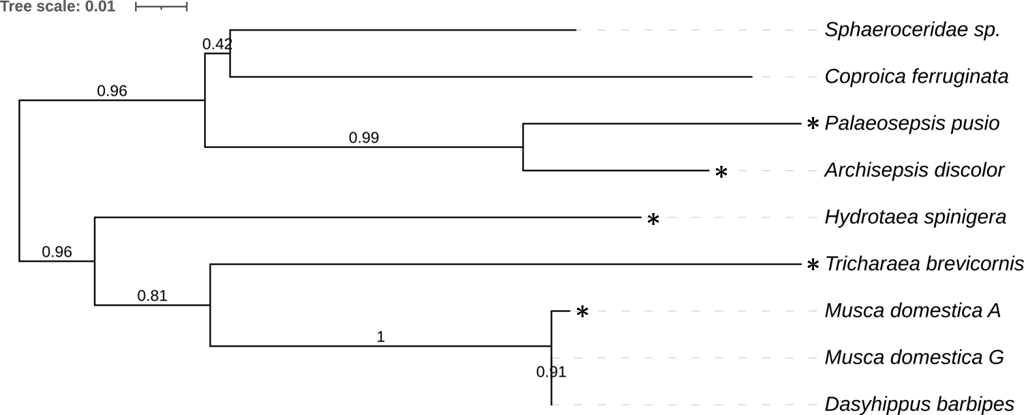


Taxonomic assignment and phylogenetic reconstruction. Statistical evaluation of branch support values using 100 bootstraps are shown. *Fly species carrying ESBL *E. coli* isolates are indicated by an asterisk.
