# Supplementary material for: The fly route of extended-spectrum-β-lactamase-producing Enterobacteriaceae dissemination in a cattle farm: from the ecosystem to the molecular scale
Source: Front Antibiot. 2024 Apr 10;3:1367936. doi: 10.3389/frabi.2024.1367936 (PMC11732033; doi:10.3389/frabi.2024.1367936)
Supplement: Supplementary file 1 [file DataSheet_1.zip › Supplementary Figure S2.DOCX]

**Supplementary Figure S2.** Maximum likelihood phylogenetic tree of the core genome of 11 *Enterobacter cloacae* complex Taxon 4 isolates from farm number 13

*
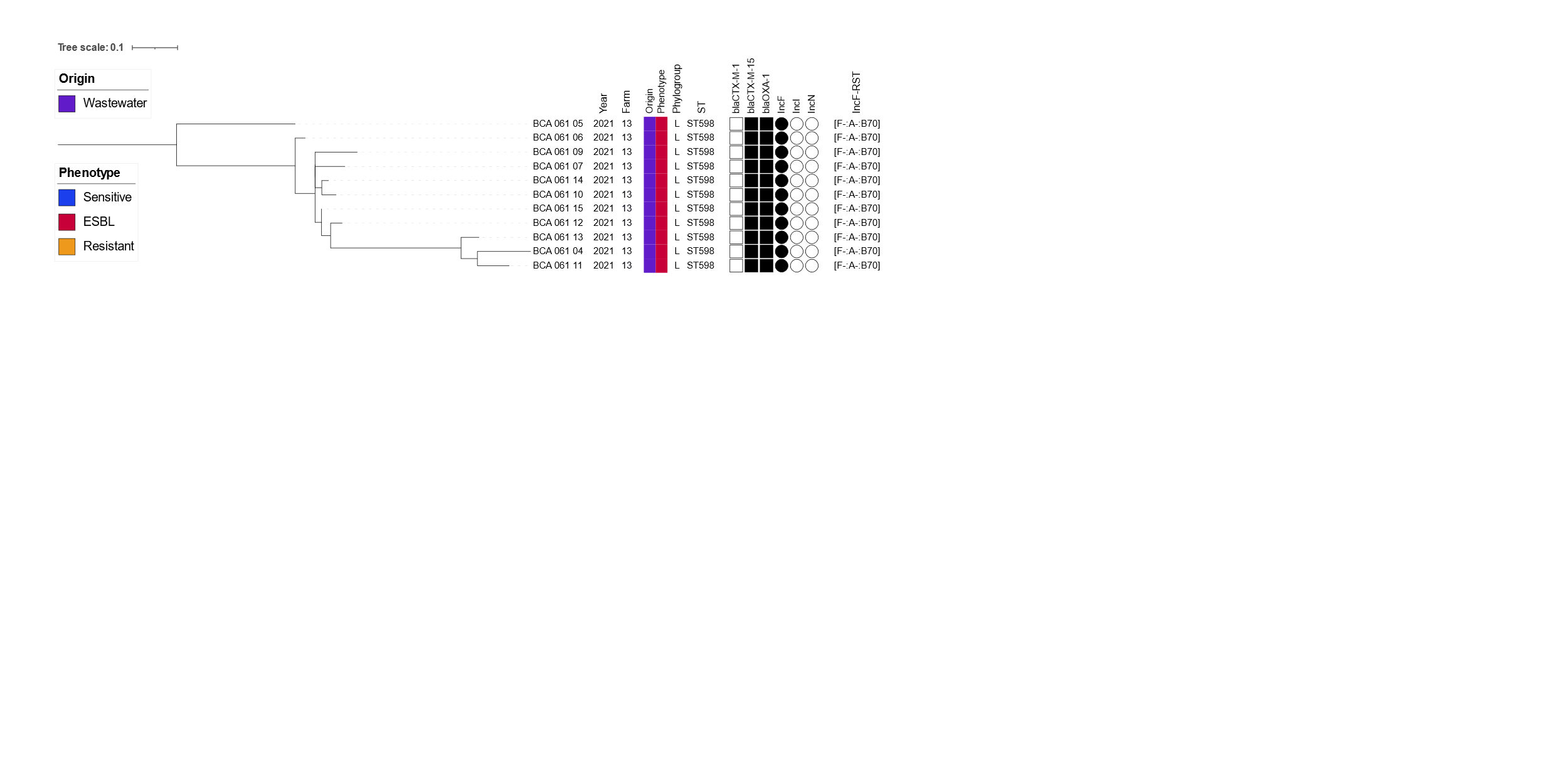
E. cloacae* phylogroups were assigned according to the Sutton clade classification (Sutton et al., 2018). Cluster ST598 represents groups with similar core genomes (≤ 23 SNP).

**Reference**

Sutton, G. G., Brinkac, L. M., Clarke, T. H., and Fouts, D. E. (2018). Enterobacter hormaechei subsp. hoffmannii subsp. nov., Enterobacter hormaechei subsp. xiangfangensis comb. nov., Enterobacter roggenkampii sp. nov., and Enterobacter muelleri is a later heterotypic synonym of Enterobacter asburiae based on computational a. *F1000Research* 7, 521. doi: 10.12688/f1000research.14566.2.
