# Supplementary material for: The fly route of extended-spectrum-β-lactamase-producing Enterobacteriaceae dissemination in a cattle farm: from the ecosystem to the molecular scale
Source: Front Antibiot. 2024 Apr 10;3:1367936. doi: 10.3389/frabi.2024.1367936 (PMC11732033; doi:10.3389/frabi.2024.1367936)
Supplement: Supplementary file 1 [file DataSheet_1.zip › Supplementary Figure S4.DOCX]

**Supplementary Figure S4.** ST3268 ESBL *E. coli* phylogeography (n=32)


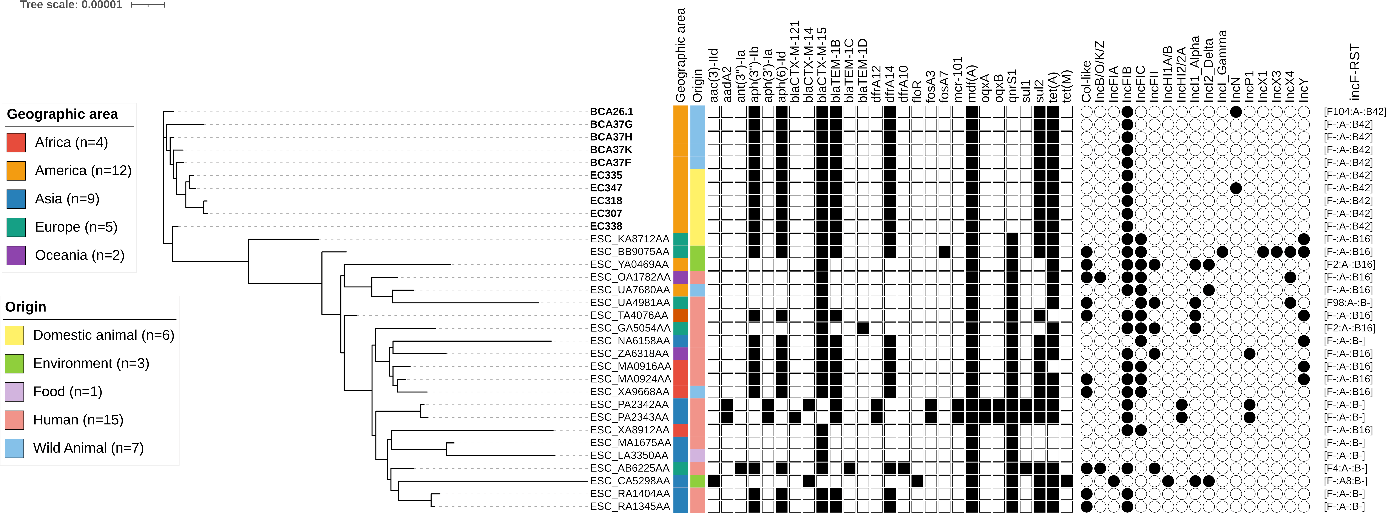


Maximum likelihood phylogenetic tree of 32 extended-spectrum β-lactamase (ESBL) enterobacterial core genomes. Geographical area and origin of the isolates are indicated by vertical colored stripes. Corresponding resistance-coding genes are indicated by black squares and plasmids by black circles. IncF, plasmid incompatibility group F; RST, Replicon Sequence Types.
