# Supplementary material for: The fly route of extended-spectrum-β-lactamase-producing Enterobacteriaceae dissemination in a cattle farm: from the ecosystem to the molecular scale
Source: Front Antibiot. 2024 Apr 10;3:1367936. doi: 10.3389/frabi.2024.1367936 (PMC11732033; doi:10.3389/frabi.2024.1367936)
Supplement: Supplementary file 1 [file DataSheet_1.zip › Supplementary Figures S3.DOCX]

**Supplementary Figures S3.**

**(a).** Syntheny of ten *bla*_CTX-M-15_/IncFIB [F-:A-:B42] *E. coli* plasmids isolated from cattle and flies


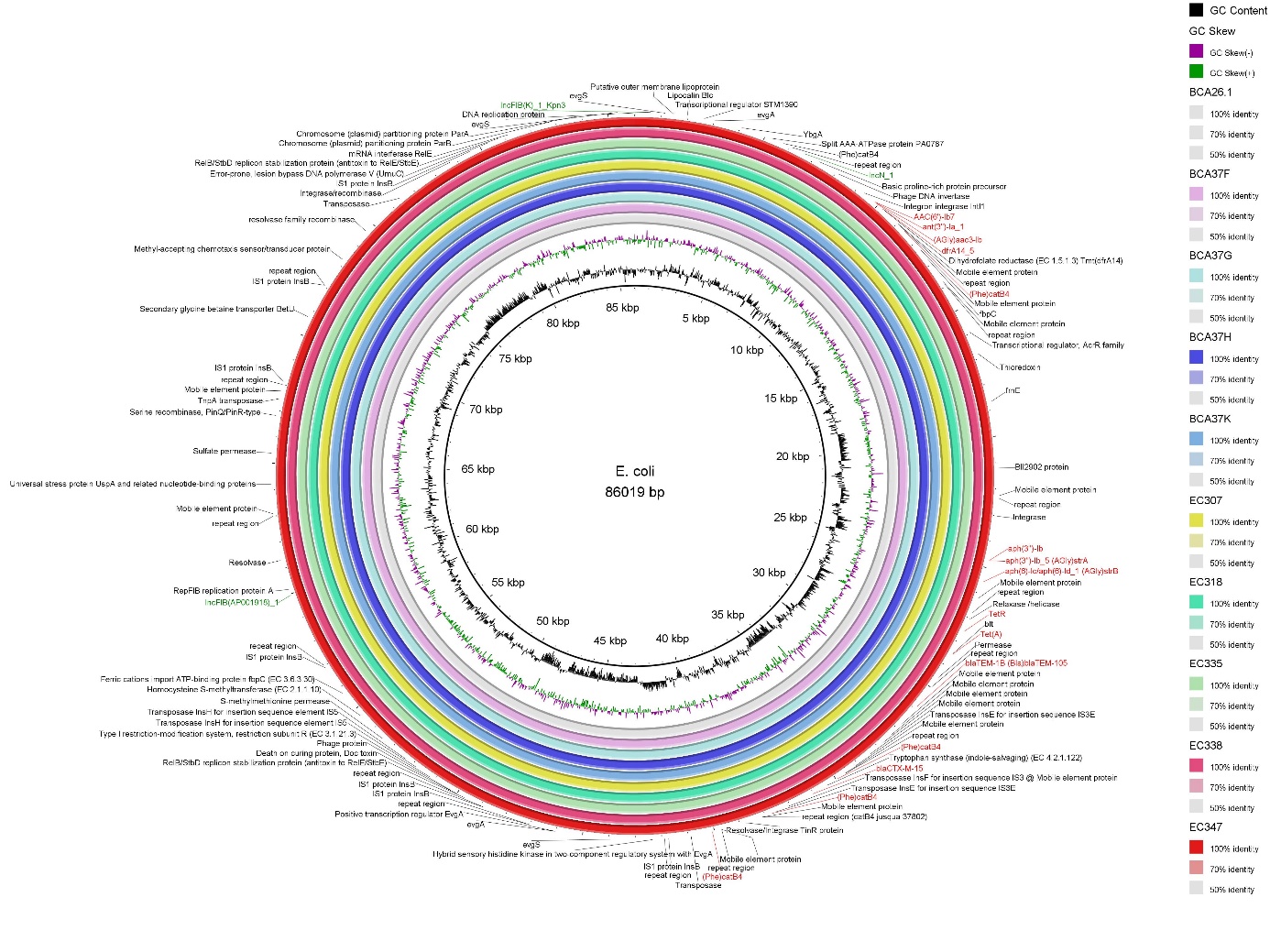
Detailed BRIG-0.95 (Alikhan et al., 2011) genome comparison visualization of the 85,190 bp non-mobile replicative multi-replicon plasmid IncFIB(K)_1_Kpn3_JN233704 (560 bp)/IncFIB(AP001918)_1_AP001918 cointegrating with a truncated IncN_1_AY046276), which harbors many mobile genetic elements (transposons, integrons, insertion sequences) and several associated resistance genes including the *bla*_CTX-M-15_. This plasmid was found in 10 ST3268 ESBL *E. coli* from cluster A (BCA26.1, BCA37F, -G, -H, -K, EC307, EC318, EC335, EC338, EC347). Annotation was performed with RAST v2.0 (Aziz et al., 2008).

**(b).** Syntheny of two IncN-pST3 *E. coli* plasmids isolated from cattle and fly


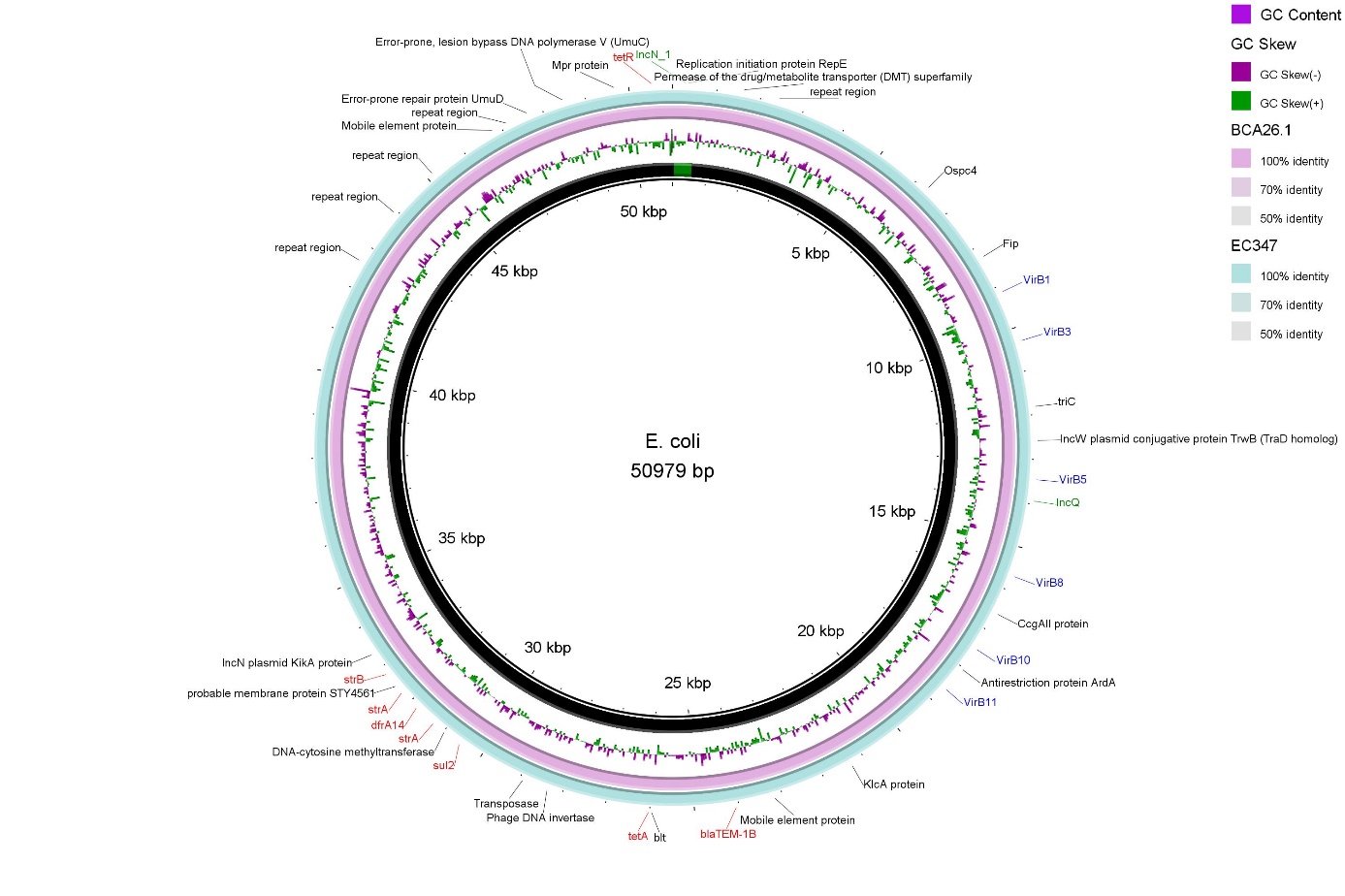
Detailed visualization of the 50,979 bp conjugative replicon plasmid (IncN_1_AY046276) by BRIG-0.95 (Alikhan et al., 2011) genome comparison. This plasmid occurs only in 2 ESBL *E. coli* of cluster ST3268.2 (26.1, EC347 from fly and cattle respectively). It carries the *bla*_TEM-1B_ gene with a cassette of resistance genes and virulence genes involved in the type IV secretion system (T4SS). Annotation was performed using RAST v2.0 (Aziz et al., 2008).

**(c).** Syntheny of nine *E. coli* phage-plasmids isolated from cattle and flies


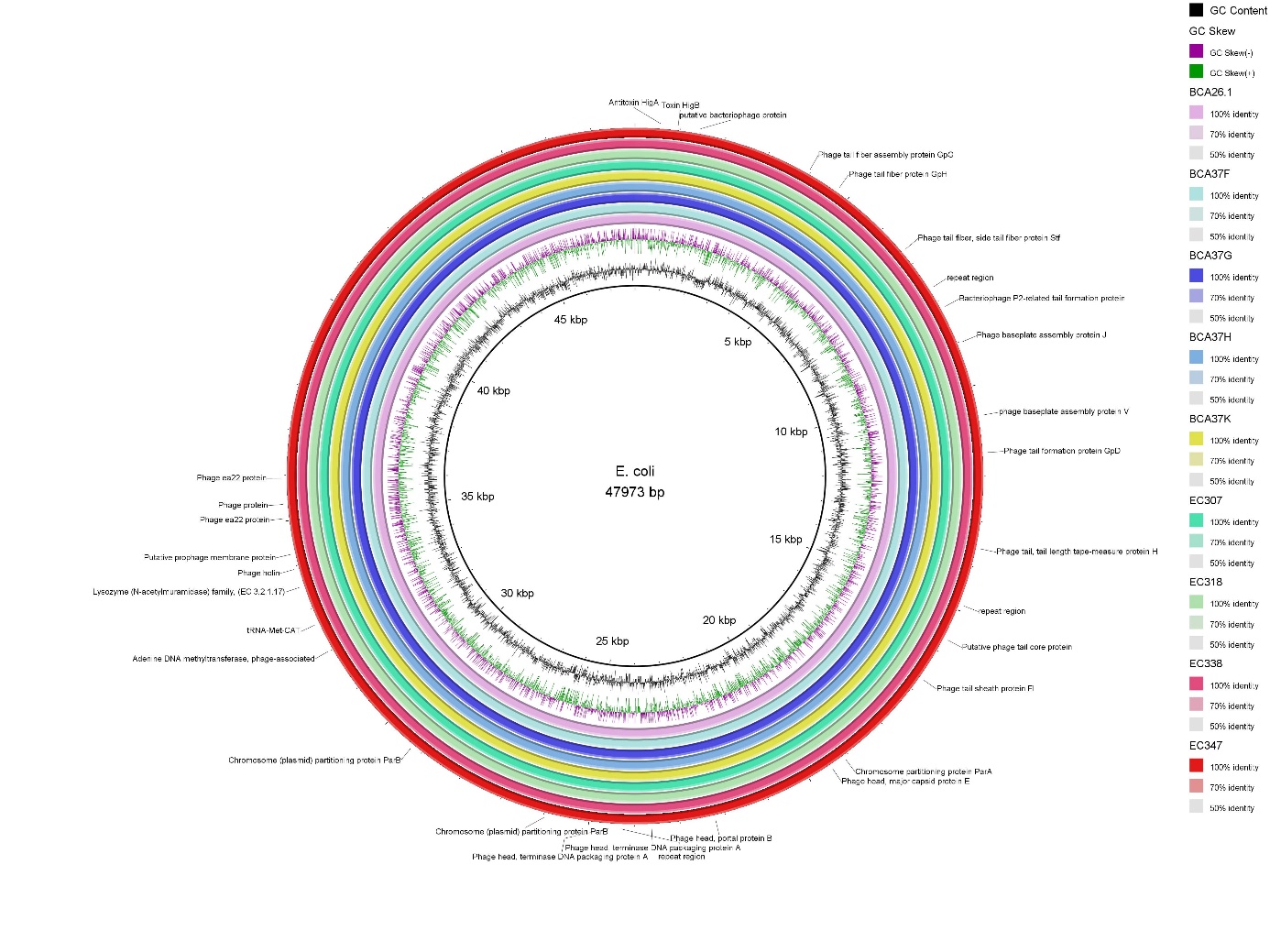
Detailed visualization of the 47,973 bp phage plasmid containing prophage regions from Vibrio and Bacillus by BRIG-0.95 (Alikhan et al., 2011) genome comparison. This plasmid carried no resistance genes and a toxin HigB/antitoxin HigA system involved in pathogenicity regulation. Annotation was performed using RAST v2.0 (Aziz et al., 2008).

**(d).** Syntheny of four *bla*_CTX-M-15_/IncFIB [F-:A-:B70] *E. cloacae* plasmids isolated from wastewater


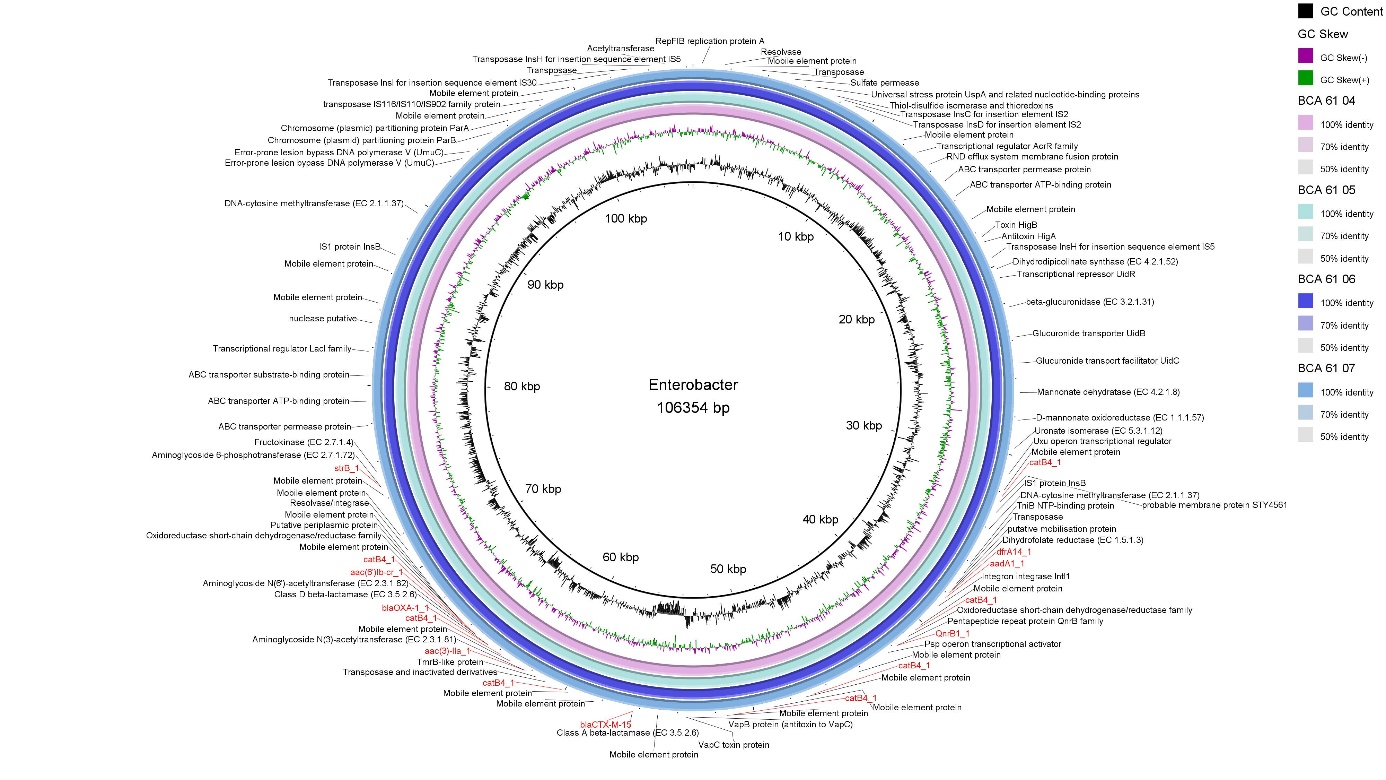
Detailed visualization of the 106,354 bp non-mobile replicative replicon plasmid IncFIB(pB171)_1_pB171 by BRIG-0.95 (Alikhan et al., 2011) genome comparison. This plasmid harbors many mobile genetic elements (transposons, integrons, insertion sequences) and several associated resistance genes including the *bla*_CTX-M-15_. This plasmid occurs in 11 ESBL *E. cloacae* complex Taxon 4 of cluster ST598 isolated from wastewater. Annotation was performed with RAST v2.0 (Aziz et al., 2008).

**References**

Alikhan, N.-F., Petty, N. K., Ben Zakour, N. L., and Beatson, S. A. (2011). BLAST Ring Image Generator (BRIG): simple prokaryote genome comparisons. *BMC Genomics* 12, 402. doi: 10.1186/1471-2164-12-402.

Aziz, R. K., Bartels, D., Best, A. A., DeJongh, M., Disz, T., Edwards, R. A., et al. (2008). The RAST Server: rapid annotations using subsystems technology. *BMC Genomics* 9, 75. doi: 10.1186/1471-2164-9-75.
