# Supplementary material for: The fly route of extended-spectrum-β-lactamase-producing Enterobacteriaceae dissemination in a cattle farm: from the ecosystem to the molecular scale
Source: Front Antibiot. 2024 Apr 10;3:1367936. doi: 10.3389/frabi.2024.1367936 (PMC11732033; doi:10.3389/frabi.2024.1367936)
Supplement: Supplementary file 1 [file DataSheet_1.zip › Supplementary Materials.DOCX]

**Supplementary Materials**

## Supplementary Material M1. bla_CTX-M-15_ gene expression

Bacterial samples were obtained from overnight cultured ESBL and non-ESBL *E. coli* in 10 mL Luria-Bertani broth media supplemented or not with oxytetracycline, ivermectin and copper at sub-inhibitory concentrations determined after MIC measurement. Bacterial density was measured with a photometer and harvested by centrifugation to adjust the concentration to 10^8^ cells/mL. Total RNA was immediately extracted using the NucleoSpin^®^ RNA Isolation Kit on a silica membrane column according to the manufacturer's recommendations (Macherey-Nagel). Total RNA concentrations were measured using the Qubit® RNA High Sensitivity Assay Kit (Thermo Fisher Scientific) on an Invitrogen Qubit™ 4.0 fluorometer (Thermo Fisher Scientific). A maximum of 2 µg of total RNA was then reverse transcribed to the corresponding cDNA using Invitrogen SuperScript™ VILO™ Master Mix (Thermo Fisher Scientific) in a total volume of 20 µL in one step as follows: 25 °C for 10 minutes, followed by 42 °C for 60 minutes and 85 °C for 5 minutes. The cDNA was then used in qPCR with TaqMan™ hydrolysis probes using the TaqMan™ Gene Expression Master Mix on a 7500 Real-Time PCR System (Thermo Fisher Scientific). *De novo* primers and probes were designed using Primer Express v3.0.1 software. The *bla*_CTX-M-15_ and 16S *rRNA* genes were amplified using the following primer and probe combinations, respectively CTXMGp1-F 5'-CGACGTTAAACACCGCCATT-3', CTXMGp1-R 5'-TGCCCGAGGTGAAGTGGTA-3', CTXMGp1-P 5'-6FAM-CGGGCGATCCGCGTG-BHQ-3', 16S-F 5'-CCAGGGCTACACGTGCTA-3', 16S-R 5'-TCTCGCGAGGTCGCTTCT-3', 16S-R 5'-6FAM-AATGGCGCATACAAA-BHQ-3'. The qPCR program was as follows 50 °C for 2 minutes, followed by 40 cycles of 10 minutes at 95 °C, 15 s at 95 °C and 1 minutes at 60 °C. RNA not subjected to RT was also included in the PCR (negative control) to ensure that positive PCR reactions were due to the presence of transcripts and not to contaminating DNA. The constitutively expressed 16S rRNA transcripts were used as a reference gene to normalize the relative level of mRNA expression. For each run, a standard curve was generated in duplicate using a 10-fold serial dilution (input range 100 ng to 10 pg) of a quantification calibrator of untreated *E. coli* cDNA (37F). The 2^‐ΔΔCT^ algorithm was used to estimate the relative expression level of *bla*_CTX-M-15_ transcripts for the two populations studied using the RQ application module on the Thermo Fisher Cloud. Each real-time PCR run included gene expression measurements of the endogenous 16S rRNA gene and the target *bla*_CTX-M-15_ gene in the corresponding samples.

**Supplementary Material M2.** Whole genome sequencing and core genome comparative analyses

DNA was extracted using a DNA Mini Kit (Qiagen). Libraries were prepared using the Nextera XT kit (Illumina) and sequencing was performed on the NextSeq 500 system (Illumina), generating 35-151bp paired-end reads for an average depth of coverage of 85-fold (minimum 78-fold, maximum 92-fold). Reads were trimmed and filtered using AlienTrimmer (Criscuolo and Brisse, 2014) v0.4.0 with an average Phred quality score of 33. Genomes were assembled using SPAdes (Bankevich et al., 2012) v3.13.0, and final quality assessed using QUAST (Gurevich et al., 2013) v5.0. Annotation of the assembled genomes was performed using Prokka (Seemann, 2014) v1.14.5, then a core genome was extracted using Roary (Page et al., 2015) v3.13.0. Maximum likelihood phylogenetic reconstruction was performed using RAxML (Stamatakis, 2014) v8.0.0 with 100 bootstrap replicates. The GTR-CAT model was used for phylogenetic reconstruction based on nucleotide sequences. The tree was plotted using iTOL (Letunic and Bork, 2021) v6.0.0. *In silico* screening and annotation of replicon plasmid types, antimicrobial resistance, virulence genes and multilocus sequence typing (MLST) were performed using a tool called catchSequenceInfo^[[1]](#footnote-1)^ v1.0.0. This tool uses ABRicate (https://github.com/tseemann/abricate) with ResFinder (Zankari et al., 2012), PlasmidFinder (Carattoli and Hasman, 2020) virulence factor database (VFDB (Chen et al., 2005)) and the multilocus sequence type (MLST) tool^[[2]](#footnote-2)^ with a threshold of 80% nucleotide identity and 80% coverage. The MLST was identified based on the corresponding Achtman MLST scheme for *E. coli* (Wirth et al., 2006). The phylogroup was identified using the EzClermont software tool (Waters et al., 2020). An in-house Perl script using the Plasmid MLST (PMLST) database (Jolley et al., 2018) and implemented in the Galaxy KaruBioNet web application^[[3]](#footnote-3)^ (Couvin et al., 2022) was used to determine specific PMLST profiles. Since ST3268 was the most represented ST in our collection of ESBL *E. coli* (10/15, 66.7%), we compared it with isolates from other countries. An Enterobase search was performed on November 29, 2022 to extract the metadata and genomic sequence of all ST3268 isolates. Only strains with complete metadata were retained, regardless of resistance phenotype (collection date, country and origin) (Zhou et al., 2020). A total of 22 sequences were obtained. The same software tools were used to characterize plasmids (Chen et al., 2005; Wirth et al., 2006; Zankari et al., 2012; Carattoli and Hasman, 2020). The phylogenetic tree was constructed as described above. Genomic identification of *Enterobacter* strains was performed using the different approaches described in our previous manuscript (Pot et al., 2022).

**Supplementary Material M3.** Multiplex long -read sequencing and hybrid assembly

MinION ligation libraries were constructed from 1 μg of unfragmented bacterial gDNA following the protocol instructions for native barcoded genomic DNA (using EXP-NBD104, EXP-NBD114 and SQK-LSK109). The final library was then mixed with sequencing buffer and loading beads and loaded onto a fresh R9.4.1 flow cell (FLO-MIN106D) according to the manufacturer's instructions and run on a laptop (MinKNOW Core v3.6.5). Single flow cell sequencing data from multiplexed barcoded isolates were run on the MinION for 48 hours. Base calling of MinION raw signals was performed using Guppy v3.2.10. Fastq files were extracted and split by barcode using the Epi2Me v3.3.0 workflow. *De novo* genome assembly was performed using a hybrid strategy on combined nanopore long reads and previously available Illumina short reads. Fully resolved assemblies were generated using the Unicycler (Wick et al., 2017) pipeline v0.4.8.0 with default parameters and visualized using Bandage (Wick et al., 2015). Quality control of nanopore data was performed using QUAST (Gurevich et al., 2013). Plasmids were aligned graphically using BRIG-0.95 (Alikhan et al., 2011), and annotated using RAST (Aziz et al., 2008) v2.0 and catchSequenceInfo. Mobilization modules characterization was performed using MOB-Suite (Robertson and Nash, 2018).

**References**

Alikhan, N.-F., Petty, N. K., Ben Zakour, N. L., and Beatson, S. A. (2011). BLAST Ring Image Generator (BRIG): simple prokaryote genome comparisons. *BMC Genomics* 12, 402. doi: 10.1186/1471-2164-12-402.

Aziz, R. K., Bartels, D., Best, A. A., DeJongh, M., Disz, T., Edwards, R. A., et al. (2008). The RAST Server: rapid annotations using subsystems technology. *BMC Genomics* 9, 75. doi: 10.1186/1471-2164-9-75.

Bankevich, A., Nurk, S., Antipov, D., Gurevich, A. A., Dvorkin, M., Kulikov, A. S., et al. (2012). SPAdes: a new genome assembly algorithm and its applications to single-cell sequencing. *J Comput Biol* 19, 455–477. doi: 10.1089/cmb.2012.0021.

Carattoli, A., and Hasman, H. (2020). PlasmidFinder and In Silico pMLST: Identification and Typing of Plasmid Replicons in Whole-Genome Sequencing (WGS). *Methods Mol. Biol.* 2075, 285–294. doi: 10.1007/978-1-4939-9877-7_20.

Chen, L., Yang, J., Yu, J., Yao, Z., Sun, L., Shen, Y., et al. (2005). VFDB: a reference database for bacterial virulence factors. *Nucleic Acids Res.* 33, D325-8. doi: 10.1093/nar/gki008.

Couvin, D., Dereeper, A., Meyer, D. F., Noroy, C., Gaete, S., Bhakkan, B., et al. (2022). KaruBioNet: a network and discussion group for a better collaboration and structuring of bioinformatics in Guadeloupe (French West Indies). *Bioinforma. Adv.* 2, vbac010. doi: 10.1093/bioadv/vbac010.

Criscuolo, A., and Brisse, S. (2014). AlienTrimmer removes adapter oligonucleotides with high sensitivity in short-insert paired-end reads. Commentary on Turner (2014) Assessment of insert sizes and adapter content in FASTQ data from NexteraXT libraries. *Front Genet* 5, 130. doi: 10.3389/fgene.2014.00130.

Gurevich, A., Saveliev, V., Vyahhi, N., and Tesler, G. (2013). QUAST: quality assessment tool for genome assemblies. *Bioinformatics* 29, 1072–1075. doi: 10.1093/bioinformatics/btt086.

Jolley, K. A., Bray, J. E., and Maiden, M. C. J. (2018). Open-access bacterial population genomics: BIGSdb software, the PubMLST.org website and their applications. *Wellcome open Res.* 3, 124. doi: 10.12688/wellcomeopenres.14826.1.

Letunic, I., and Bork, P. (2021). Interactive Tree Of Life (iTOL) v5: an online tool for phylogenetic tree display and annotation. *Nucleic Acids Res.* 49, W293–W296. doi: 10.1093/nar/gkab301.

Page, A. J., Cummins, C. A., Hunt, M., Wong, V. K., Reuter, S., Holden, M. T., et al. (2015). Roary: rapid large-scale prokaryote pan genome analysis. *Bioinformatics* 31, 3691–3693. doi: btv421 [pii]10.1093/bioinformatics/btv421.

Pot, M., Reynaud, Y., Couvin, D., Dereeper, A., Ferdinand, S., Bastian, S., et al. (2022). Emergence of a Novel Lineage and Wide Spread of a *bla*(CTX-M-15)/IncHI2/ST1 Plasmid among Nosocomial Enterobacter in Guadeloupe. *Antibiot. (Basel, Switzerland)* 11. doi: 10.3390/antibiotics11101443.

Robertson, J., and Nash, J. H. E. (2018). MOB-suite: software tools for clustering, reconstruction and typing of plasmids from draft assemblies. *Microb. genomics* 4. doi: 10.1099/mgen.0.000206.

Seemann, T. (2014). Prokka: rapid prokaryotic genome annotation. *Bioinformatics* 30, 2068–2069. doi: btu153 [pii]10.1093/bioinformatics/btu153.

Stamatakis, A. (2014). RAxML version 8: a tool for phylogenetic analysis and post-analysis of large phylogenies. *Bioinformatics* 30, 1312–1313. doi: 10.1093/bioinformatics/btu033.

Waters, N. R., Abram, F., Brennan, F., Holmes, A., and Pritchard, L. (2020). Easy phylotyping of *Escherichia coli* via the EzClermont web app and command-line tool. *Access Microbiol.* 2. doi: https://doi.org/10.1099/acmi.0.000143.

Wick, R. R., Judd, L. M., Gorrie, C. L., and Holt, K. E. (2017). Completing bacterial genome assemblies with multiplex MinION sequencing. *Microb. Genomics* 3. doi: https://doi.org/10.1099/mgen.0.000132.

Wick, R. R., Schultz, M. B., Zobel, J., and Holt, K. E. (2015). Bandage: interactive visualization of de novo genome assemblies. *Bioinformatics* 31, 3350–3352. doi: 10.1093/bioinformatics/btv383.

Wirth, T., Falush, D., Lan, R., Colles, F., Mensa, P., Wieler, L. H., et al. (2006). Sex and virulence in *Escherichia coli*: an evolutionary perspective. *Mol Microbiol* 60, 1136–1151. doi: MMI5172 [pii] 10.1111/j.1365-2958.2006.05172.x.

Zankari, E., Hasman, H., Cosentino, S., Vestergaard, M., Rasmussen, S., Lund, O., et al. (2012). Identification of acquired antimicrobial resistance genes. *J Antimicrob Chemother* 67, 2640–2644. doi: dks261 [pii]10.1093/jac/dks261.

Zhou, Z., Alikhan, N.-F., Mohamed, K., Fan, Y., and Achtman, M. (2020). The EnteroBase user’s guide, with case studies on Salmonella transmissions,Yersinia pestis phylogeny, and Escherichia core genomic diversity. *Genome Res.* 30, 138–152. doi: 10.1101/gr.251678.119.

1. <https://github.com/karubiotools/galaxy_ipg_tools> [↑](#footnote-ref-1)
2. (<https://github.com/tseemann/mlst>) [↑](#footnote-ref-2)
3. (<http://calamar.univ-ag.fr/c3i/galaxy_karubionet.html> [↑](#footnote-ref-3)
